# Metamorphosis Reverses the Behavioral Phenotype in *Rana arvalis* Along a Latitudinal Gradient

**DOI:** 10.1002/ece3.71945

**Published:** 2025-08-13

**Authors:** Maria Cortazar‐Chinarro, Alberto Corral‐Lopez, Deike U. Lüdtke, Fredrik Tegnér, Emilien Luquet, Anssi Laurila

**Affiliations:** ^1^ Department of Ecology and Genetics Uppsala University Uppsala Sweden; ^2^ ISOE—Institute for Social‐Ecological Research Frankfurt Germany; ^3^ Senckenberg Biodiversity and Climate Research Centre Frankfurt/Main Germany; ^4^ Université Claude Bernard Lyon 1 LEHNA UMR 5023, CNRS, ENTPE Villeurbanne France; ^5^ Institut Universitaire de France (IUF) Paris France

## Abstract

Understanding how demographic processes and environmental conditions shape behavioral variation across populations is pivotal in evolutionary ecology. However, the role that such processes play in the link between behavior and life‐history traits across populations remains largely unclear. The moor frog ( *Rana arvalis* ) has colonized Sweden via two distinct routes: from the south via Denmark and from the north via Finland. We collected *R. arvalis* eggs from multiple populations along a 1,700 km latitudinal gradient across Northern Europe and raised tadpoles in a common garden experiment. We assessed developmental growth and proactivity levels in ca. 300 individuals at two key stages of anuran larval development: tadpoles (Gosner stage 32) and froglets (Gosner stage 46). We found strong behavioral differences along the latitudinal gradient and between developmental stages. Tadpoles from northernmost populations were bolder (shorter time to leave a shelter) and showed higher activity levels in an open field test compared to those from southern populations. However, these behavioral patterns reversed at the froglet stage, individuals from northern populations showing reduced proactivity compared to those from southern populations. Further analyses indicated significant associations between developmental growth and boldness, with contrasting patterns across developmental stages and colonization routes. These findings support recent revisitations of the pace‐of‐life syndrome theory, emphasizing a decoupling of correlations between behavior and life‐history traits across ontogeny, likely reflecting adaptive responses to divergent ecological and demographic constraints along the latitudinal gradient rather than a single fast‐slow continuum.

## Graphical abstract

Investigating behavior and life history traits in moor frog across a broad latitudinal gradient, we reveal a striking reversal in proactive behavioral tendencies between the tadpole and froglet developmental stages. Furthermore, we demonstrate that the relationship between proactivity and growth rates is shaped by developmental stage, local adaptation, and colonization history, providing new insights into the ecological and evolutionary drivers of behavioral variation.

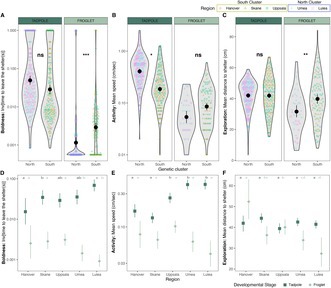

## 1 Introduction

Individuals within a species often show consistent differences in behavioral and life‐history traits, such as boldness, activity level, or growth rate (Healy et al. 2019; Stearns 1992; Wilson et al. 1994). These traits are frequently functionally and evolutionarily linked, forming the base of the Pace‐Of‐Life Syndrome (POLS) hypothesis, which explains how behavioral tendencies coevolve with specific physiological and life‐history traits, including metabolic rate, fecundity, and growth (Biro and Stamps 2008; Careau et al. 2008; Réale et al. 2010; Stamps 2007). These behavioral tendencies are typically described along a proactive–reactive continuum, where proactive individuals tend to be bolder and more aggressive, while reactive individuals are generally more cautious and flexible (Réale et al. 2010). According to the POLS hypothesis, these covariations arise from trade‐offs in energy allocation. For example, individuals investing heavily in fast growth and early reproduction may do so at the cost of reduced behavioral flexibility. Such trade‐offs often result in consistent interindividual differences in behavior across time and contexts, commonly referred to as animal personality (Bakker 1986; Bouchard and Loehlin 2001; Groothuis and Carere 2005; Wilson et al. 1994). The POLS framework has provided valuable insight into how animal personalities may constrain individual responses to environmental variation, such as perceived risks, availability of resources, or the social conditions (e.g., Spiegel et al. 2015; Villegas‐Ríos et al. 2018), and has been particularly successful in explaining interspecific patterns (e.g., Healy et al. 2019; Van de Walle et al. 2023). However, mixed empirical evidence at the within‐species level suggests the need to integrate additional factors, such as species‐specific demographic history and ecological pressures, to better understand the links between behavior, physiology, and life history (e.g., Chang et al. 2024; Gopal et al. 2023; Montiglio et al. 2018).

Latitudinal gradients represent one of the most important biogeographic patterns on Earth (Fischer 1960). Populations at higher latitudes face harsher climatic conditions and shorter breeding seasons, environmental pressures that also influence the composition of predator and parasite communities, as well as the intensity of biotic interactions (Willig et al. 2003; Poulin and Leung 2011; Schemske et al. 2009; Roslin et al. 2017). These challenging conditions have driven the evolution of life‐history strategies that differ markedly from those typically found at lower latitudes (Stearns 1992). According to the POLS framework, such broad environmental variation leads to systematic differences in behavior and life‐history along latitudinal gradients, with populations at higher latitudes showing a faster pace of life, often linked to more risk‐prone and bold tendencies (proactive behaviors; Foster et al. 2015; Gerlai and Csányi 1990; Higgins et al. 2022; Mitchell et al. 1977). For example, in many anuran tadpoles, the more demanding conditions at high altitudes (lower temperature, shorter breeding seasons) favor faster growth and development rates to ensure a successful metamorphic transition in a limited timeframe (Berven and Gill 1983; Laugen et al. 2003; Orizaola et al. 2010; Luquet et al. 2019). This accelerated development is associated with higher foraging activity, associated with a faster pace of life, and is potentially enabled by reduced predator densities at high latitudes (Laurila et al. 2006, 2008), thus creating a link between environmental pressures, behavior, and life history in line with POLS predictions.

Similar relationships between environmental context and pace‐of‐life traits have been documented in anurans, such as in comparisons between island and continental populations (Brodin et al. 2013), or along altitudinal gradients (Luquet et al. 2015). However, other studies challenge this pattern, indicating a complex relationship between latitude and personality traits. For example, eastern mosquitofish ( *Gambusia holbrooki* ) populations have shown reduced boldness at higher latitudes, despite experiencing lower predation pressure (Culumber 2022). This suggests that reduced ecological risk alone may not always select for proactive traits, and that local adaptation, population history, or energy trade‐offs may influence behavioral evolution in more complex ways. To address these inconsistencies and better understand how geographical variation influences behavior and life‐history strategies, additional integrative studies are needed.

Most investigations of POLS focus on behavioral traits measured at a single life stage (Cabrera et al. 2021). However, animal personality may shift across developmental stages, particularly in response to changes in ecological conditions across ontogeny. This ontogenetic variation in personality should be of particular importance in organisms that show complex life cycles, such as amphibians. These species exhibit a dramatic metamorphosis, involving substantial changes in morphology, physiology, and behavior as aquatic larvae transition into terrestrial juveniles (Cohen 1985; Wilbur 1980). Consequently, amphibian larvae and juveniles vary greatly in many ecological aspects, for example, by occupying distinct trophic niches (Kelleher et al. 2018; Wilbur 1980). Most anuran larvae are herbivores or omnivores, whereas post‐metamorphic individuals are carnivores, with dispersal and reproduction occurring at this stage (Wilbur 1980). The shift of habitat at metamorphosis may also lead to substantial changes in predation pressure (Alford 1999). Under the POLS framework, such strong ecological and functional contrasts across life stages could select for stage‐specific behaviors, potentially disrupting correlations between traits across ontogenetic stages in amphibians ‐a phenomenon referred to as behavioral syndrome decoupling (Sih et al. 2004). Previous studies on this topic have produced mixed results. Some report that species with complex life cycles show behavioral consistency across stages (Bégué et al. 2024; Brodin 2009; Wilson and Krause 2012), while others find absent or weak relationships between individual behavioral traits, such as proactivity, measured before and after metamorphosis (Amat et al. 2018; Brodin et al. 2013; Monceau et al. 2017; Plaskonka Płaskonka et al. 2024). This discrepancy between theoretical works and empirical results may result from the lack of consideration of the relationship between personality and life history according to ecological conditions. Consequently, understanding the variation of behavior in the proactive‐reactive continuum across developmental stages in amphibian populations and how they associate with life history across multiple ecological conditions is crucial to resolve this apparent discrepancy.

North European populations of the moor frog ( *Rana arvalis* ) provide a suitable model for investigating evolutionary processes shaping behavioral phenotypes and life‐history traits across diverse ecological conditions. This species has a wide latitudinal distribution with well‐documented variation in life history traits across its geographical range (see, e.g., Laurila et al. 2006; Luquet et al. 2019; Räsänen et al. 2008). Following the last glaciation, the species colonized the Scandinavian peninsula through two distinct routes: one from the north through present‐day Finland and another from the south, with a contact zone established in northern central Sweden (Cortázar‐Chinarro et al. 2017; Knopp and Merilä 2009). These postglacial colonization events and subsequent demographic processes have significantly influenced the divergence between populations. Luquet et al. (2019) demonstrated strong divergence between southern and northern lineages, with strong selection pressures driving higher growth, development rates, and larger body size in northern populations. Additionally, genetic analyses revealed clear differentiation between two major genetic clusters corresponding to northern and southern populations (Cortázar‐Chinarro et al. 2017; Meyer‐Lucht et al. 2019; Rödin‐Mörch et al. 2019). These findings suggest that *Rana arvalis* populations offer a valuable natural system to examine how historical and ecological variation shape the co‐evolution of behavioral and life‐history traits across a latitudinal gradient and these two genetic clusters.

In this study, we evaluate behavioral variation across populations, regions, and genetic clusters of *R. arvalis* at two developmental stages. Specifically, we conducted a common garden experiment using populations from five different regions in northern Europe and performed repeated open field tests on individuals before and after metamorphosis to quantify behavioral traits on the proactive–reactive continuum. These data were combined with life‐history measurements to provide insight into the relationship between behavior, development, and life history across large‐scale geographical variation. In *R. arvalis* , shorter breeding seasons and harsher climatic conditions at higher latitudes are associated with faster development (Luquet et al. 2019; Meyer‐Lucht et al. 2019). Based on the POLS framework, we hypothesized that:

- Proactive behaviors would be stronger in high latitudes at early developmental stages, reflecting adaptation to time‐constrained environments;
- Behavioral traits expressed along the proactive–reactive continuum would differ between larval and post‐metamorphic stages, due to stage‐specific ecological challenges and selective pressures;
- Proactive behaviors would be positively associated with faster developmental growth across the geographical range.

## 2 Methods

### 2.1 Sample Collection and Latitudinal Gradient

**FIGURE 1:**
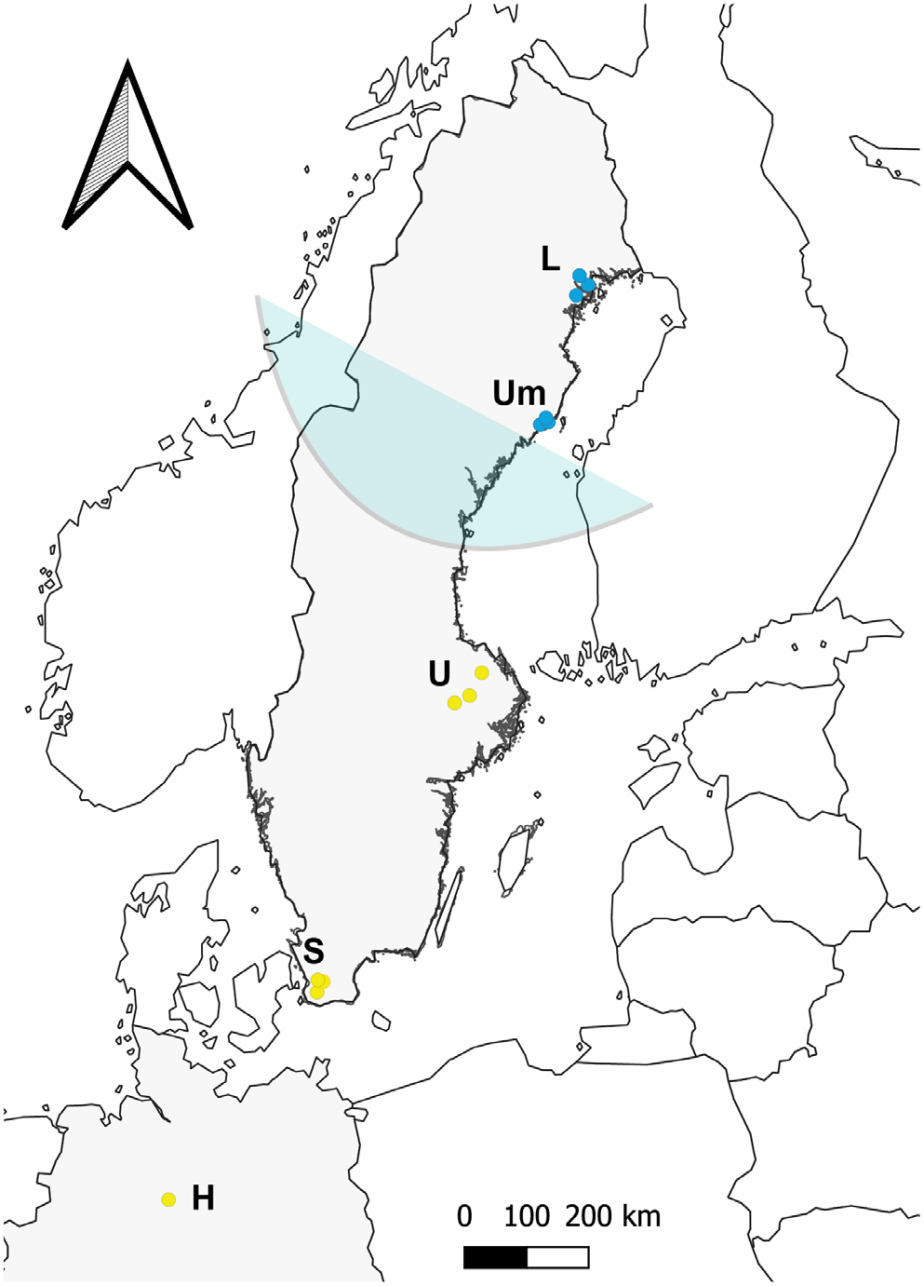
**Study sampling.** Geographic location of the five *Rana arvalis* populations sampled along a latitudinal gradient in northern Europe: Hanover (H), Skåne (S), Uppsala (U), Umeå (Um), and Luleå (L). Yellow dots represent populations belonging to the southern genetic cluster, while blue dots represent populations from the northern genetic cluster. The shaded area marks the approximate contact zone between the two postglacial colonization routes into Scandinavia.

*Rana arvalis* has a broad Eurasian distribution, from the North Sea coast to western Siberia as it inhabits a wide range of open shallow wetlands (e.g., swamps, meadows), spawning clutches in shallow stagnant waters, each containing 500–3000 eggs deposited usually in one clump. (Sillero et al. 2014). We collected *R. arvalis* eggs in five regions along a 1700 km latitudinal gradient from northern Germany (Hanover) to northern Sweden (Luleå), covering a large part of the latitudinal distribution of the species (see Luquet et al. 2019; Figure 1). A sample of 50–100 fertilized eggs from each of 10 freshly laid clutches (hereafter families) was collected at each of three breeding populations in each region, except Hanover, where eggs were collected from only one site (13 populations in total; Luquet et al. 2019). The distance between populations within the same region varied from 8 to 50 km (average 20 km), allowing us to consider that they were different genetic units (see Luquet et al. 2019; Rödin‐Mörch et al. 2019). The timing of the breeding season along the latitudinal gradient varies from mid‐March (Hanover) to late May (Luleå). The egg masses were transported in local pond water to the laboratory and then kept in a climate room at 16°C and 16 L:8D photoperiod in isolation in 5 L aquaria filled with aerated water until they hatched and reached Gosner stage 25 (independent feeding; Gosner 1960).

### 2.2 Experimental Design

#### 2.2.1 Common Garden Experiment

The tadpoles were reared in a common garden experiment to compare behavior differences between two life stages: tadpoles and juveniles (see Luquet et al. 2019 for further details). At Gosner stage 25, in which tadpole size is very homogeneous within a clutch, three tadpoles from each of the ten families per population were randomly selected without regard to size. Tadpoles were individually raised in 0.75 L opaque plastic vials (roughly cuboid, ca. 10 × 10 × 10 cm) until metamorphosis. They were fed chopped spinach *ad libitum*, and the water was changed every third day with aerated water ensuring high oxygen concentration (8–10 mg/L). The experiment was carried out in climate‐controlled walk‐in rooms with an average water temperature of 16°C, corresponding to a temperature commonly encountered in the natural environment. The photoperiod was 16 L:8D, corresponding to the natural day length experienced in May at Uppsala (located approximately at the centre of the present gradient). When the tadpoles reached Gosner stage 32 (Gosner 1960), we performed the set of behavioral assays at tadpole stage (see below), weighed them to the nearest 0.1 mg with an electronic scale, and returned them to their vials until they reached metamorphosis (emergence of the first forelimb; stage 42, Gosner 1960). At metamorphosis, the water was reduced to form a gentle slope allowing the metamorphs to emerge without drowning, and the vials were closed with a lid to prevent the animals from escaping. On the day when the metamorphs reached the froglet stage (i.e., Gosner stage 46, tail entirely resorbed), we performed a second set of behavioral assays and weighed them to the nearest 0.1 mg.

#### 2.2.2 Recording of Behavior

We recorded the behavior of tadpoles and froglets (*n* tadpole/froglet: Hanover = 31/20, Skåne = 89/65, Uppsala = 90/73, Umeå = 89/65, Luleå = 90/61, total = 389/284) individually using an open field test in circular arenas (diameter = 90 cm). The arenas contained a small circular plastic shelter (diameter = 5 cm) placed at the center. This opaque, dome‐shaped structure allowed individuals to hide underneath and included a trapdoor that could be gently lifted remotely using a string (individuals could not exit this structure until we lifted the trapdoor). Trials were conducted in a room maintained at 16°C. For tadpole tests, the arenas were filled with 10 cm of water, while for froglet tests, the arena floor was moist. Each individual was placed in the shelter and allowed to acclimate for 10 minutes. Behavioral recording started as we lifted the shelter trapdoor, allowing individuals to voluntarily exit and explore the arena. Videos were recorded using a digital video camera (Sony, HDR‐HC1E Handy Camera) placed 1.70 m above the experimental arena.

To assess the degree to which individuals maintained consistency in proactivity over repeated measurements, a representative subset of individuals was tested twice. A total of 30 tadpoles and 33 froglets (*n* tadpole/froglet: Hanover = 2/2, Skåne = 7/6, Uppsala = 6/8, Umeå = 6/8, Luleå = 9/9) were re‐assayed 24–48 h after their first assay, during which time they were returned to their housing vials. To minimize potential habituation to the experimental setup effects, froglets were not tested twice if they had been tested twice as tadpoles. All individuals were tested during the froglet stage, regardless of their testing history as tadpoles.

#### 2.2.3 Data Acquisition

Using the recordings, we scored emergence time from the shelter, a widely used proxy for *boldness* (Mazué et al. 2015), by scoring the time point when the tadpole or froglet had completely emerged from the shelter provided. In the cases where the individual did not leave the shelter, the trial was stopped after 20 min for tadpoles (*n* = 6) and 30 min for froglets (*n* = 126).

For individuals that left the shelter, we analyzed 5 min of recording to measure proactivity based on two additional traits: (i) *activity*, obtained as the mean speed during the recording, and (ii) *exploration*, obtained as the average distance to the shelter during the recorded time. Videos were analyzed using the computer vision software Ctrax v0.5.19 (Branson et al. 2009). The quality of the tracking was checked and manually corrected using the software's graphical user interface, which flags and enables correction of tracking errors based on improbable changes in speed and trajectory. We then collapsed the frame‐by‐frame positional data obtained and calculated the behavioral proxies using the tracker package (https://github.com/Ax3man/trackr) in Rv4.1.3 (R Core Team 2024).

### 2.3 Statistical Analysis

#### 2.3.1 Repeatability of Individual Behavior Within Developmental Stages

As we were unable to obtain repeated measurements of activity or exploration for froglets that did not leave the shelter during the first or second trial (*n* = 17), we used data on time to leave the shelter in our representative subset of individuals tested twice to assess individual repeatability of boldness. Repeatability, which quantifies behavioral consistency by estimating the proportion of total variance attributable to differences among individuals (Dingemanse and Dochtermann 2013; Nakagawa and Schielzeth 2010) was calculated within a linear mixed model (LMM) framework. Specifically, we evaluated how much of the variance in boldness was explained by individual identity based on repeated assays. Separate Gaussian models with log‐transformed emergence time as the dependent variable were run for tadpoles and froglets to quantify the proportion of variance in boldness explained by individual identity across repeated trials. Each model included region as a fixed effect to account for broad‐scale environmental differences across the latitudinal gradient that may influence behavior, and population as a random effect to account for potential non‐independence of individuals sampled from the same pond. Analyses were conducted using the ‘rptR’ package in Rv4.4.1 (Stoffel et al. 2017; R Core Team 2024).

#### 2.3.2 Behavioral Traits Across the Latitudinal Gradient

We evaluated possible differences in behavior between geographic regions, genetic clusters, and developmental stages using LMMs implemented in the package ‘lme4’ in Rv4.4.1 (Bates et al. 2015; R Core Team 2024). Regions refer to the five geographic sampling locations along the latitudinal gradient, while genetic clusters refer to separated northern and southern lineages containing these regions and that had been identified in prior genetic analyses (northern lineage: Luleå, Umeå regions; southern lineage: Uppsala, Skåne, and Hanover regions; Cortázar‐Chinarro et al. 2017; Figure 1). The Uppsala region is located near the contact zone between northern and southern genetic clusters, typically showing more intermediate patterns (Luquet et al. 2019; Rödin‐Mörch et al. 2019). We modeled region and cluster effects separately to independently assess the effect of postglacial colonization history and ecological contexts within regions.

To test for differences between regions, we included developmental stage, region, and their interaction as fixed effects, with population of origin and individual identity as random intercept effects. To test for differences between genetic clusters, we included developmental stage, genetic cluster, and their interaction as fixed effects, with population of origin, region, and individual identity as random intercept effects. Individual mass at the time of testing was included as a covariate in all models. Model fit and significance were assessed using conditional F tests with the Kenward‐Roger approximation for the degrees of freedom using the ‘pbkrtest’ and ‘lmerTest’ packages (Halekoh and Højsgaard 2014; Kuznetsova et al. 2017). To assess differences between regions, we obtained post hoc contrasts of the best‐fitted model using the ‘emmeans’ package and including false discovery rate correction for multiple tests (Lenth 2016). The significance of random effects was tested using likelihood ratio tests comparing models with or without random effects in the full fixed effect structure.

A large number of froglets did not leave the shelter and were assigned an emergence value of the decided cutoff point (1800 s; *n* = 126). Therefore, the data on time to emerge from shelter (boldness) were analyzed using a mixed‐effect survival test performed with the 'coxme' package (Therneau and Lumley 2015). The model structure was analogous to the LMMs above. The significance of fixed and random effects was tested using likelihood ratio tests comparing models with or without the tested effect in the complete structure of fixed effects.

To assess whether individual behavioral tendencies were consistent across metamorphosis, we estimated the repeatability of each behavior across developmental stages using LMMs analogous to those described above in the R package ‘rptR’ in Rv4.4.1 (Stoffel et al. 2017; R Core Team 2024). These models were based on repeated measurements of the same individuals, tested once as tadpoles and again as froglets. Each model included individual identity as a random effect, allowing us to partition variance and evaluate whether behavioral differences between individuals persisted across ontogeny. Developmental stage was included as a fixed effect to account for overall mean differences in behavior between the two stages. This analysis represents a distinct set of models from the within‐stage repeatability estimates described earlier, as it specifically addresses the consistency of all behavioral traits measured across the metamorphic boundary.

#### 2.3.3 Link Between Behavior and Life History Across Genetic Clusters

We combined data on the time to reach metamorphosis (Gosner stage 42, in days) and the mass at the same stage to calculate developmental growth (mass/time at Gosner stage 42) for the individuals evaluated for their behavior. This trait integrates developmental rate and body size, providing an individual‐level life‐history metric. As this data represents a subset of individuals included in previous studies on the quantitative differentiation of life history traits along the latitudinal gradient (Luquet et al. 2019; Meyer‐Lucht et al. 2019), we did not focus on comparing population‐level variation in the current study. Instead, we used this variable to assess how life‐history correlates with individual behavioral variation.

To test whether behavioral traits predict growth rate, and whether this relationship is influenced by genetic cluster, geographical region, or developmental stage, we built LMMs with growth rate as the dependent variable. Behavioral traits (i.e., boldness/activity/exploration, analyzed independently), genetic cluster or region, and developmental stage were included as fixed effects, with population of origin and individual identity included as random intercept effects. Model fit and significance were assessed as previously through conditional F tests with the Kenward‐Roger approximation for the degrees of freedom using ‘pbkrtest’' and ‘lmerTest’ packages (Halekoh and Højsgaard 2014; Kuznetsova et al. 2017). We obtained post hoc contrasts of fitted models using the 'emmeans' package including false discovery rate correction for multiple tests (Lenth 2016). The significance of random effects was tested using likelihood ratio tests comparing models with or without random effects in the full fixed effect structure.

## 3 Results

### 3.1 Repeatability of Emergence Time (Boldness)

**FIGURE 2:**
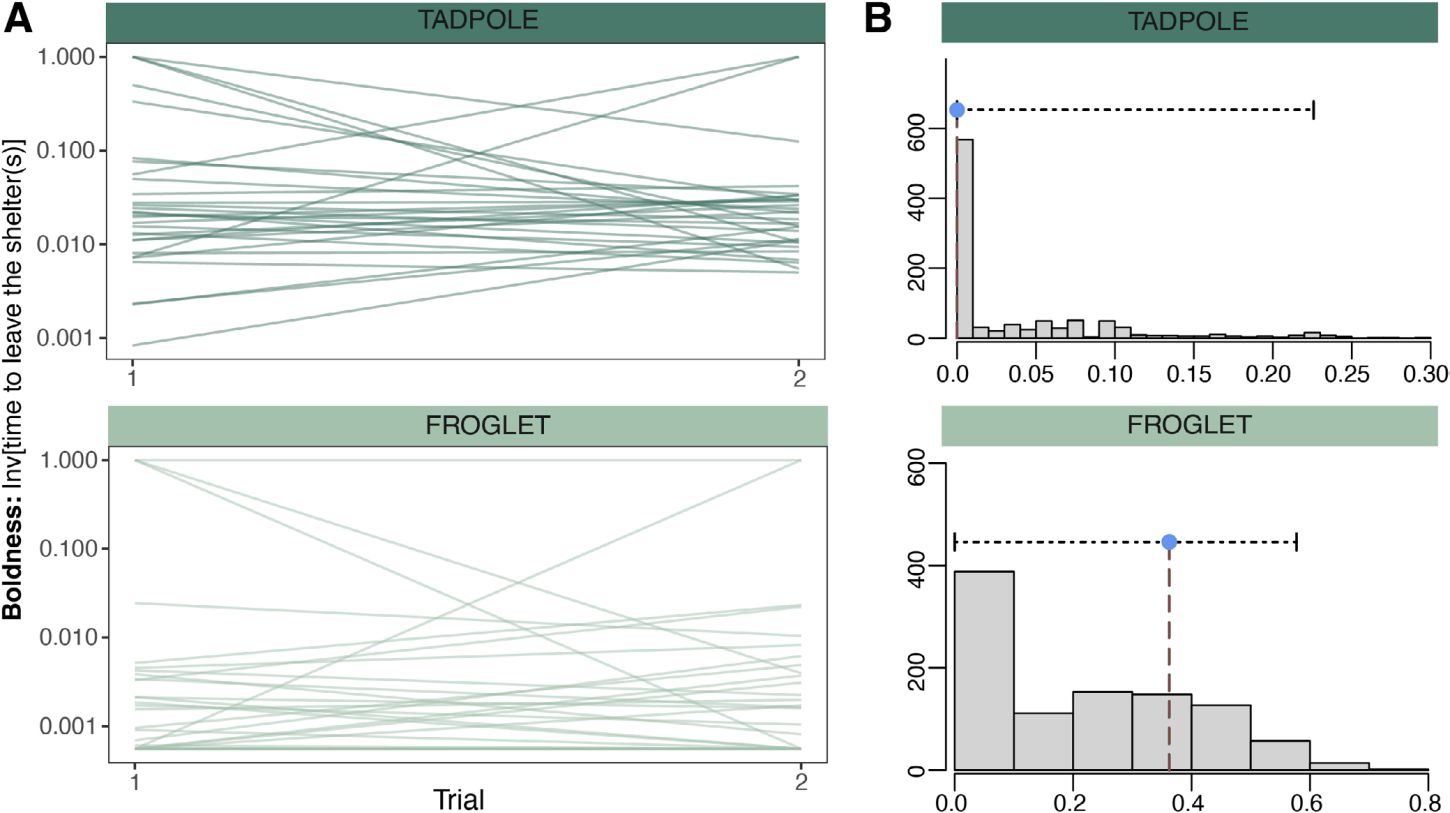
**Consistency of individual behavior.** (A) Individual differences in measurements of time to emerge from the shelter taken during two independent trials performed to moor frog tadpoles (*n* = 30, dark green) and froglets (*n* = 33, light green) raised in a common garden experiment sampled across multiple regions of a 1700 km latitudinal gradient in northern Europe. (B) Distribution of adjusted repeatability estimates of an individual time to emerge from the shelter in bootstrapping tests using a linear mixed model framework after controlling for effects introduced by region (see Stoffel et al. 2017). Blue dot indicates mean adjusted repeatability with confidence intervals for time to emerge in tadpoles (top) and froglets (bottom).

Our repeatability estimations of time to emerge from the shelter (controlling for the effects of region) indicated that individuals measured twice had repeatable emergence times between trials at the froglet stage, but not at the tadpole stage (Tadpole: ∆ log emergence time = 1.48 ± 0.27 SE; *R* = 0.000 ± 0.060 SE, *p* = 1, Froglet: ∆ log emergence time = 1.24 ± 0.34 SE; *R* = 0.362 ± 0.189 SE, *p* = 0.023; Figure 2; Table 1).

**TABLE 1:** Consistency of individual behavior.

Developmental stage	Factor	_adj_ *R*/*R* ^2^	SE	95% CI	*p*
Tadpole	Individual	0.00	0.06	[0, 0.226]	1.00
	Population	0.00	0.06	[0, 0.131]	1.00
	Fixed (region)	0.23	0.16	[0.088, 0.683]	—
Froglet	Individual	0.36	0.19	[0, 0.577]	0.023*
	Population	0.00	0.18	[0, 0.535]	1.00
	Fixed (region)	0.24	0.18	[0.079, 0.769]	—

*Note:* Results for repeatability analyses of measurements of time to emerge from a shelter taken twice to moor frogs during the tadpole stage (*n* = 30), and the froglet stage (*n* = 33) raised in a common garden experiment following collection from multiple regions across a 1700 km latitudinal gradient in northern Europe. Using a linear mixed model framework, we estimated adjusted repeatability of boldness for individual and population after controlling for effects introduced by region in the model (_adj_ *R*; see Stoffel et al. 2017), as well as the quantification of uncertainty for the variance explained by region of origin (*R* ^2^ coefficient, see Stoffel et al. 2017). * indicates *p* < 0.05.

### 3.2 Behavior Across Genetic Clusters, Regions, and Developmental Stages

**FIGURE 3:**
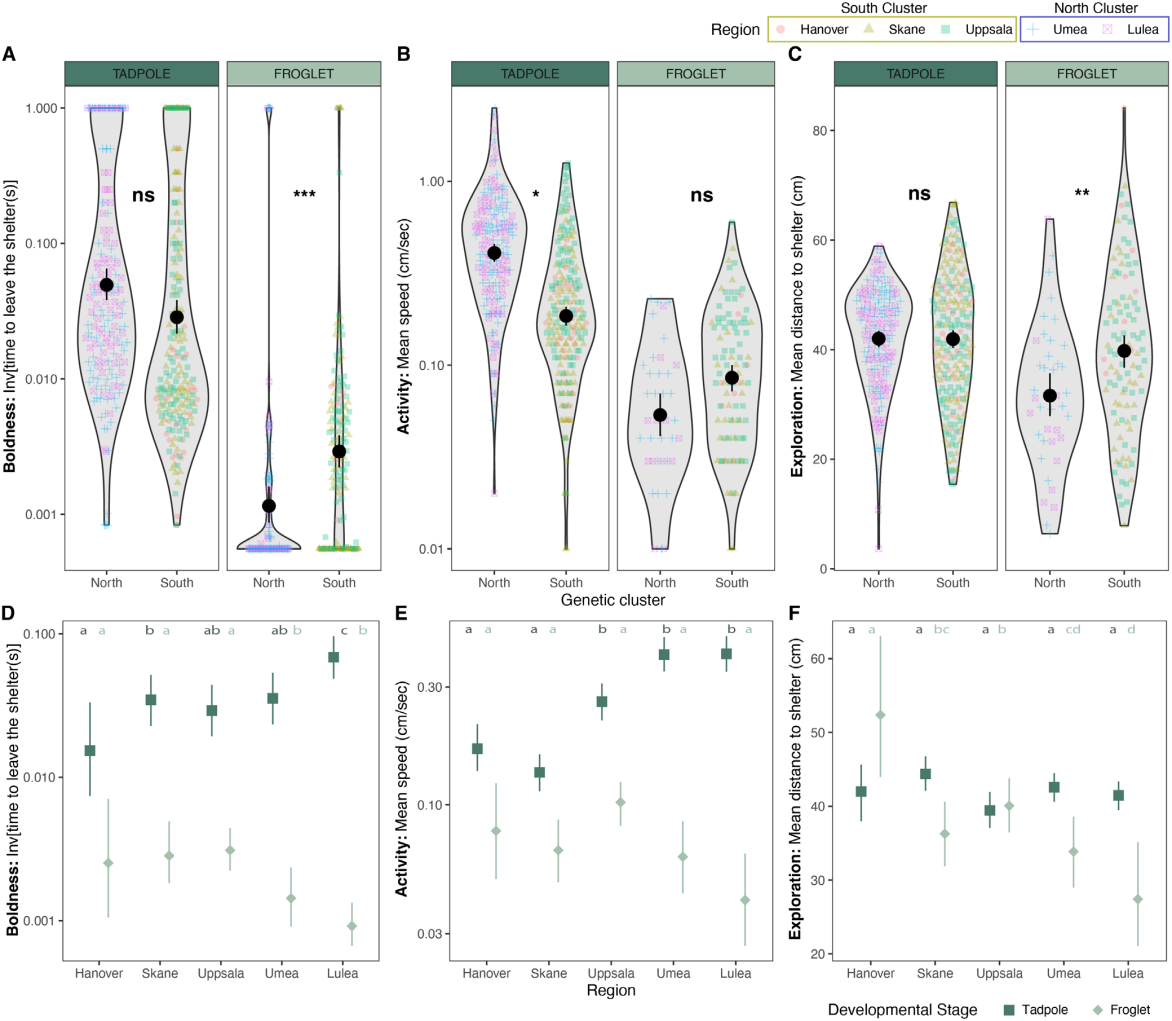
**Behavior of moor frogs across the latitudinal gradient.** Boldness (A), activity (B) and exploration (C) measured in moor frog tadpoles (dark green) and froglets (light green) across a 1700 km latitudinal gradient and raised in a common garden experiment (South genetic cluster: Hanover (Germany), red dot; Skåne (Sweden), yellow triangle; Uppsala (Sweden), green square; North genetic cluster: Umeå (Sweden), blue cross; Luleå (Sweden), pink crossed square). Text indicates *p*‐values of significance tests comparing the difference in behavior between genetic clusters (see Table 2; ns: *P* > 0.05, *p < 0.05, **p < 0.01, ***p < 0.001). Mean value and standard error of boldness (D), activity (E), and exploration (F) of tadpoles (dark green squares) and froglets (light green diamonds) for the five regions sampled. Average values for regions not sharing any letter are significantly different (*p* < 0.05) in post hoc contrasts of independent models assessing differences in behavior across regions for tadpoles (dark green letters) and froglets (light green letters; see Table S1).

Boldness—There was a significant interaction between developmental stage and genetic cluster on boldness (mean time to emerge from the shelter (s) ± SE: South tadpoles: 121 ± 11.9; North tadpoles: 81 ± 14.5; South froglets: 807 ± 60.0; North froglets: 1402 ± 56.1; Figure 3; Table 2). Specifically, boldness did not differ between tadpoles from the South and North genetic clusters, but froglets from the South populations were bolder (longer time to emerge) than froglets from the North populations (Tadpole_North vs. South_: ∆ ratio = 1.26 ± 0.21 SE, *z* = 1.40, *p* = 0.16, Froglet_North vs. South_: ∆ ratio = 0.50 ± 0.08 SE, *z* = −4.47, *p* < 0.001; Figure 3A). Similarly, statistical models assessing the effect of geographic region indicated a significant interaction effect between the developmental stage and region on boldness (mean time to emerge from the shelter (s) ± SE: *Tadpoles*—Hanover: 209 ± 49.1; Skåne: 99 ± 13.2; Uppsala: 113 ± 17.2; Umeå: 124 ± 27.8; Luleå: 39 ± 6.1; *Froglets*—Hanover: 1095 ± 185.0; Skåne: 870 ± 96.3; Uppsala: 672 ± 80.9; Umeå: 1290 ± 82.9; Luleå: 1521 ± 72.6; Figure 3D; Table 3). Specifically, tadpoles from the northernmost region (Luleå) were significantly bolder than those from all other regions sampled, including Umeå, which belongs to the same genetic cluster (∆ ratio [SE]: Hanover‐Luleå: 0.38 [0.09]—adj*p* < 0.001; Skåne‐Luleå: 0.65 [0.12]—adj*p* = 0.040; Uppsala‐Luleå: 0.55 [0.09]—adj*p* = 0.001; Umeå‐Luleå: 0.51 [0.08]—adj*p* < 0.001; Figure 3D; Table S1). The tadpoles from the southernmost region (Hanover) also tended to be shyer (i.e., took a longer time to emerge from the shelter) compared to other populations. This difference was only significant in the comparison to Skåne (∆ ratio [SE]: 0.58 [0.13]—adj*p* = 0.040; Figure 3D; Table S1), while comparisons with Uppsala and Umeå were not significant (Table S1). In froglets, individuals from the two northern regions were significantly shyer than individuals from the rest of the regions studied in the gradient (Figure 2D; see Table S1). The random effect of population was never significant for our statistical analyses of boldness (Tables 2 and 3 for models that incorporate genetic clusters or regions, respectively). Emergence time was not repeatable in measurements taken for individuals at both tadpole and froglet stages (*R* = 0.079 ± 0.056 SE, *p* = 0.10; Figure S1), indicating individuals did not maintain consistent boldness levels across developmental stages.

**TABLE 2:** Behavior across genetic clusters and developmental stages.

Boldness: time to leave the shelter					Activity: mean speed				Exploration: mean distance to shelter			
Predictors	Δ log‐lik	*χ* ^2^	*p*	df	SumSq (mean sq)	*F*‐value	*p*	df (Num_dem_)	SumSq (mean sq)	*F*‐value	*p*	df (Num_dem_)
Cluster	16.0***	32.04	**< 0.001**	1	0.04 (0.04)	1.33	0.32	1_3.23_	785.1 (785.1)	6.02	0.064	1_4.42_
Stage	−31.5***	137.03	**< 0.001**	1	1.96*** (1.96)	61.07	**< 0.001**	1_466.5_	716.5* (716.5)	5.50	**0.019**	1_412.8_
Mass at recording	2.6*	5.22	**0.022**	1	0.14* (0.13)	4.23	**0.040**	1_504.3_	157.0 (157.0)	1.21	0.27	1_409.7_
Cluster × stage	14.5***	28.98	**< 0.001**	1	1.10*** (1.10)	34.26	**< 0.001**	1_310.6_	1164.6** (1164.6)	8.94	**0.003**	1_339.9_

Random effects	Δ log‐lik	*χ* ^2^	*p*	df	Δ log‐lik	*χ* ^2^	*p*	df	Δ log‐lik	*χ* ^2^	*p*	df
Id	0.0	0.00	0.97	1	−1.4	2.77	0.096	1	0.0	1.81	0.18	1
Population	0.8	1.66	0.19	1	3.0*	6.07	**0.013**	1	0.0	0.0	1	1
Region	0.0	0.05	0.81	1	0.2	0.55	0.45	1	0.0	0.13	0.71	1
*σ* ^2^					0.03				130.28			
*τ*_00_	0.00_id_				0.01_id_				14.62_id_			
	0.01_population_				0.00_population_				0.00_population_			
	0.00_region_				0.00_region_				2.09_region_			
Observations	655				512				512			
Marginal *R* ^2^	0.71				0.365				0.058			

*Note:* Results for statistical analyses evaluating behavioral traits across tadpole and froglet stages in moor frogs originated from five different regions across a 1700 latitudinal gradient and raised in a common garden experiment (South genetic cluster: Hanover (Germany), Skåne (Sweden), Uppsala), North genetic cluster: Umeå (Sweden), Luleå (Sweden). Boldness data was analyzed using a mixed‐effect survival model, while activity and exploration data was analyzed using linear mixed models. Full models included genetic cluster, developmental stage and interactions as fixed effects, while population of origin, region and individual identity as random intercept effects. Individual mass at the time of recording was added as a covariate.

* *p* < 0.05.

** *p* < 0.01.

*** *p* < 0.001.

**TABLE 3:** Behavior across regions and developmental stages.

Predictors	Boldness: time to leave the shelter				Activity: mean speed				Exploration: mean distance to shelter			
	Δ log‐lik	*χ* ^2^	*p*	df	SumSq (mean sq)	*F*‐value	*p*	df (Num_dem_)	SumSq (mean sq)	*F*‐value	*p*	df (Num_dem_)
Region	29.2*	58.58	**< 0.001**	1	0.23 (0.06)	1.80	0.20	4_11.1_	2606.1** (651.5)	5.23	**0.007**	4_16.5_
Stage	78.9***	156.29	**< 0.001**	1	1.53*** (1.53)	48.05	**< 0.001**	1_453.4_	204.2 (204.2)	1.65	0.20	1_455.2_
Mass at recording	2.3*	4.73	**0.030**	1	0.12* (0.12)	3.91	**0.048**	1_500.4_	163.7 (163.7)	1.32	0.25	1_450.1_
Region × stage	24.3***	48.66	**< 0.001**	1	1.24*** (0.31)	9.71	**< 0.001**	4_288.0_	3511.7*** (877.7)	7.09	**< 0.001**	4_309.7_

Random effects	Δ log‐lik	*χ* ^2^	*p*	df	Δ log‐lik	*χ* ^2^	*p*	df	Δ log‐lik	*χ* ^2^	*p*	df
Id	0.0	0.00	0.95	1	1.6	3.25	0.071	1	−0.8	2.40	0.12	1
Population	0.0	0.08	0.77	1	1.4	2.74	0.098	1	0.0	0.00	1	1
*σ* ^2^					0.03				123.83			
*τ*_00_	0.00_id_				0.01_id_				16.85_id_			
	0.00_population_				0.00_population_				0.00_population_			
Observations	655				512				512			
Marginal *R* ^2^	0.725				0.389				0.104			

*Note:* Results for statistical analyses evaluating behavioral traits across tadpole and froglet stages in moor frogs originated from five different regions across a 1700 latitudinal gradient and were raised in a common garden experiment (Hanover (Germany), Skåne (Sweden), Uppsala, Umeå (Sweden), Luleå (Sweden)). Boldness data were analyzed using a mixed‐effect survival model, while activity and exploration data were analyzed using linear mixed models. Full models included developmental stage, region, and their interaction as fixed effects, while population of origin, and individual identity as random intercept effects. Individual mass at the time of recording was added as a covariate.

* *p* < 0.05.

** *p* < 0.01.

*** *p* < 0.001.

Activity—There was a significant interaction between developmental stage and genetic cluster, and developmental stage and region in activity (Tables 2 and 3). At the genetic cluster level, tadpoles from the South populations were less active than those from the North populations, but there was no difference in the froglet development stage (mean speed (cm/s) ± SE: South tadpoles: 0.26 ± 0.02; North tadpoles: 0.53 ± 0.03; South froglets: 0.11 ± 0.01; North froglets: 0.07 ± 0.01; *Tadpole*_North vs. South_: ∆ estimate (sqrt) = 0.18 ± 0.05 SE, *t* = 3.41, *p* = 0.036, Froglet_North vs. South_: ∆ estimate (sqrt) = −0.06 ± 0.06 SE, *t* = −0.98, *p* = 0.36; Figure 3B). At the regional level, the activity of tadpoles differed markedly among geographical regions sampled, while froglets had activity levels that were more similar across regions (Mean speed (cm/s) ± SE: *Tadpoles*—Hanover: 0.20 ± 0.02; Skåne: 0.18 ± 0.01; Uppsala: 0.36 ± 0.03; Umeå: 0.53 ± 0.04; Luleå: 0.53 ± 0.04. *Froglets*—Hanover: 0.10 ± 0.02; Skåne: 0.09 ± 0.01; Uppsala: 0.13 ± 0.01; Umeå: 0.08 ± 0.01; Luleå: 0.05 ± 0.01; Figure 3). Tadpoles from the two southern regions (Hanover, Skåne) had lower activity than tadpoles from the intermediate latitude (Uppsala region) and northern regions (Umeå, Luleå). The activity of tadpoles from the intermediate region (Uppsala) was marginally lower than those from northern regions (Figure 3E; see Table S1). The random effect of population on activity was significant in the genetic cluster model (Table 2) and only marginal in the region model (Table 3), indicating that a substantial portion of variation in activity occurred at the population level, independent of broader genetic cluster or geographic regional effects. Activity was significantly repeatable in measurements taken for individuals at both the tadpole and froglet stage (*R* = 0.158 ± 0.078 SE, *p* = 0.034; Figure S1).

Exploration—There was a significant interaction between developmental stage and genetic cluster or region in exploration (Tables 2 and 3). Exploration of tadpoles from the South and North genetic clusters was similar, while froglets from the North genetic cluster showed reduced exploration compared to those from the Southern cluster (mean distance from shelter (cm) ± SE: South tadpoles: 41.9 ± 0.71; North tadpoles: 42.0 ± 0.83; South froglets: 39.8 ± 1.20; North froglets: 31.6 ± 1.17; Tadpole_North vs. South_: ∆ estimate = −1.07 ± 2.09 SE, *t* = −0.51, *p* = 0.63, Froglet_North vs. South_: ∆ estimate = −9.03 ± 2.76 SE, *t* = −3.27, *p* = 0.005; Figure 3C). In contrast to observations for activity levels, exploration of froglets differed strongly, while tadpoles showed more similar exploration across regions (mean distance to shelter (cm) ± SE: *Tadpoles*—Hanover: 42.0 ± 1.9; Skåne: 44.4 ± 1.3; Uppsala: 39.4 ± 1.2; Umeå: 42.6 ± 1.0; Luleå: 41.5 ± 1.0. *Froglets*—Hanover: 52.4 ± 3.5; Skåne: 36.3 ± 1.8; Uppsala: 40.1 ± 1.7; Umeå: 33.8 ± 1.6; Luleå: 27.4 ± 1.7; Figure 3). Specifically, for the froglet stage, the exploration of the southernmost region (Hanover) was higher than all other regions along the gradient (Figure 3F; Table S1). Froglets from the two northernmost regions (Luleå and Umeå) had significantly lower exploration than froglets from the southern regions (except between Skåne and Umeå; Figure 3F, Table S1). The random population effect was never significant (Tables 2 and 3 for models incorporating genetic clusters or regions, respectively). Exploration was not repeatable in measurements taken for individuals at both tadpole and froglet stages (*R* = 0.099 ± 0.078 SE, *p* = 0.13; Figure S1).

### 3.3 Influence of Behavioral Traits on Growth Rate

**FIGURE 4:**
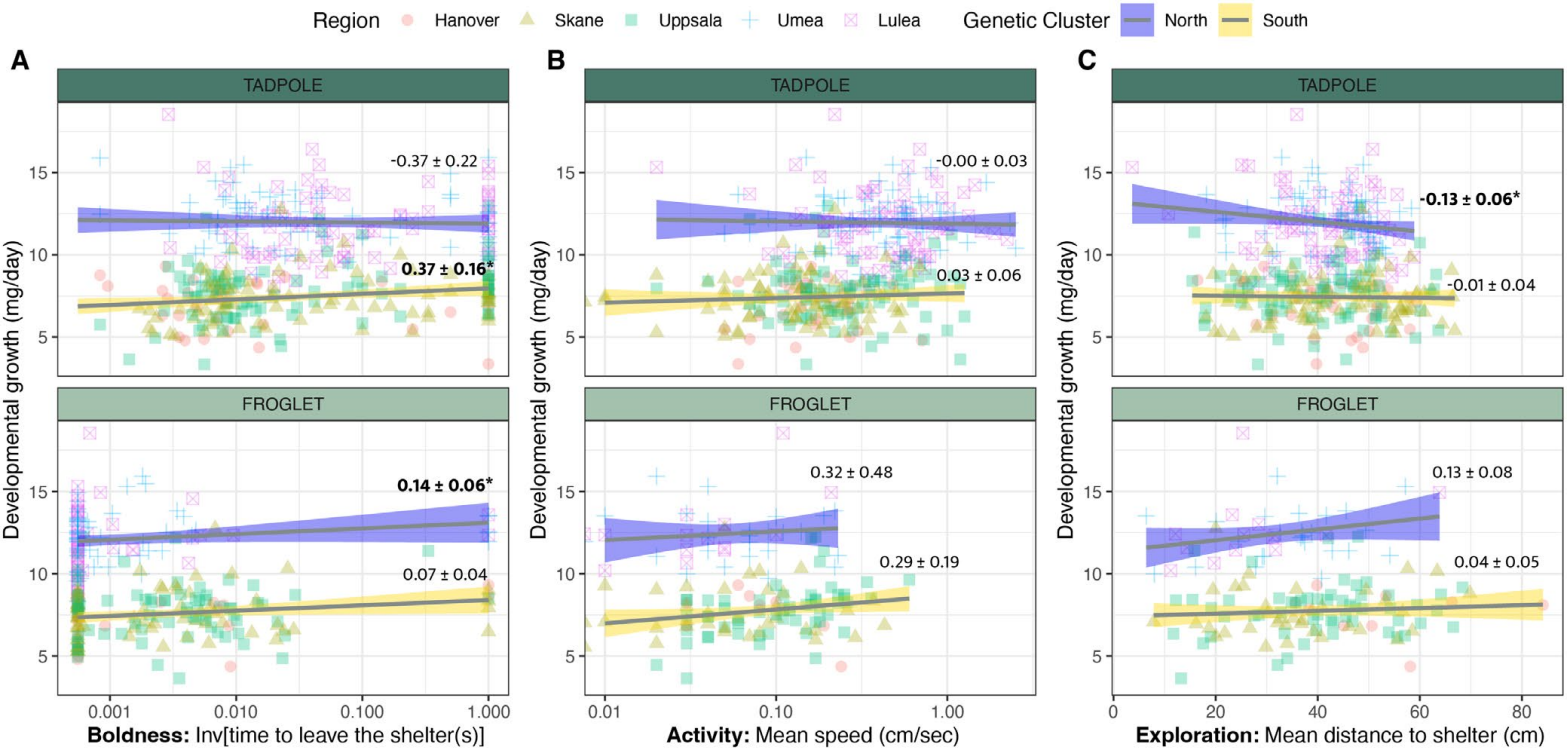
**Association between behavior and life history in moor frogs across the latitudinal gradient.** Relationships between (A) boldness, (B) activity, and (C) exploration and developmental growth (mass/time at Gosner stage 42) in moor frog tadpoles (top row) and froglets (bottom row) sampled across a 1700 km latitudinal gradient and raised in a common garden experiment (South genetic cluster: Hanover (Germany), red dot; Skåne (Sweden), yellow triangle; Uppsala (Sweden), green square; North genetic cluster: Umeå (Sweden), blue cross; Luleå (Sweden), pink crossed square). Trend lines and confidence intervals are displayed for the relationship between each behavior and developmental growth for values at the North (blue) and South (yellow) genetic clusters. Text indicates estimated marginal means and standard error for each trend in post hoc contrasts of statistical models (*p* < 0.05 in bold; see Table S2).

Growth/boldness—We found a significant three‐way interaction between boldness, genetic cluster, and developmental stage on growth rate (Figure 4A; Table 4). In the southern cluster, tadpoles that emerged more quickly from the shelter (i.e., bolder individuals) showed faster growth rates. This relationship was not found in northern tadpoles (Tadpole_North vs. South_: ∆ estimate = 0.74 ± 0.28 SE, *p* = 0.008; Figure 4A; Table S2). This pattern was reversed at the froglet stage, with bolder individuals in the northern cluster showing faster growth, whereas no association was found in the southern cluster froglets (Figure 4A; Table S2).

**TABLE 4:** Influence of behavior on life history (growth rate) across genetic clusters and developmental stages.

Growth rate including boldness (time to leave the shelter) as a predictor				
Predictors	SumSq (mean sq)	*F*‐value	*p*	df (Num_dem_)
Boldness	0.18 (0.18)	0.57	0.45	1_620.5_
Cluster	67.65 (67.65)***	205.0	**< 0.001**	1_41.8_
Stage	0.44 (0.44)	1.35	0.25	1_618.4_
Boldness × cluster	1.79 (1.79)**	5.44	**0.020**	1_620.4_
Boldness × stage	0.17 (0.17)	0.52	0.48	1_620.3_
Cluster × stage	0.74 (0.74)	2.24	0.13	1_618.4_
Boldness × cluster × stage	2.63 (2.63)**	7.96	**0.004**	1_620.3_

Random effects	Δ log‐lik	*χ* ^2^	*p*	df
Population	38.0***	16.1	**< 0.001**	1
Region	0.0	0	1	1
*σ* ^2^	0.33			
ICC	0.06			
Observations	631			
Marginal *R* ^2^	0.647			

Growth rate including activity (mean speed) as predictor				
Predictors	SumSq (mean sq)	*F*‐value	*p*	df (Num_dem_)
Activity	0.49 (0.49)	1.47	0.22	1_475.2_
Cluster	21.09 (21.09)***	62.36	**< 0.001**	1_166.5_
Stage	0.88 (0.88)	2.62	0.11	1_476.2_
Activity × cluster	0.00 (0.00)	0.00	0.98	1_475.2_
Activity × stage	0.42 (0.42)	1.25	0.27	1_474.0_
Cluster × stage	0.04 (0.04)	0.11	0.74	1_476.1_
Activity × cluster × stage	0.07 (0.07)	0.02	0.89	1_474.0_

Random effects	Δ log‐lik	*χ* ^2^	*p*	df
Population	6.1***	11.5	**< 0.001**	1
Region	0.0	0	1	1
*σ* ^2^	0.34			
ICC	0.06			
Observations	489			
Marginal *R* ^2^	0.631			

Growth rate including exploration (mean distance to shelter) as predictor				
Predictors	SumSq (mean sq)	*F*‐value	*p*	df (Num_dem_)
Exploration	0.02 (0.02)	0.07	0.80	1_474.2_
Cluster	77.23 (77.23)***	230.52	**< 0.001**	1_16.5_
Stage	1.68 (1.68)*	5.02	**0.025**	1_479.1_
Exploration × cluster	0.01 (0.01)	0.04	0.84	1_474.2_
Exploration × stage	2.12 (2.12)*	6.34	**0.01**	1_475.8_
Cluster × stage	0.37 (0.37)	1.10	0.29	1_479.1_
Exploration × cluster × stage	0.95 (0.95)	2.84	0.092	1_475.8_

Random effects	Δ log‐lik	*χ* ^2^	*p*	df
Population	7.7***	12.5	**< 0.001**	1
Region	0.0	0	1	1
*σ* ^2^	0.34			
ICC	0.07			
Observations	489			
Marginal *R* ^2^	0.634			

*Note:* Results for statistical analyses evaluating growth rate in moor frog tadpoles and froglets originating from five different regions across a 1700 latitudinal gradient and raised in a common garden experiment (South genetic cluster: Hanover (Germany), Skåne (Sweden), Uppsala), North genetic cluster: Umeå (Sweden), Luleå (Sweden). Growth rate linear mixed models included the behavior of interest, genetic cluster, developmental stage, and their interaction as predictors, and population of origin and region as random intercept effects.

* *p* < 0.05.

** *p* < 0.01.

*** *p* < 0.001.

When assessing the effect of geographical region, the boldness‐growth relationship also varied by developmental stage, with a significant three‐way interaction between boldness, region, and stage (Figure S2A; Table 5; Table S3). This was primarily driven by a positive association between growth rate and boldness in tadpoles sampled in Uppsala (estimate_Tadpole_ = 0.79 ± 0.26 SE, *p* = 0.027) and Skåne (estimate_Tadpole_ = 0.89 ± 0.35 SE, *p* = 0.059), in contrast to the absence of this relationship in other regions (Table S4; Figure S2A). Furthermore, this pattern differed across developmental stages, with positive associations observed in tadpoles from Uppsala and Skåne, but not in froglets from these regions (Uppsala: ∆ estimate_Tadpole vs. Froglet_ = 0.79 ± 0.27 SE; *t* = 2.91, *p* = 0.004; Skåne: ∆ estimate_Tadpole vs. Froglet_ = 0.77 ± 0.36 SE, *t* = 2.16, *p* = 0.031; Figure S2A; Table S5). The random effect of population was significant in both genetic cluster and region statistical models, indicating population‐level variation in the boldness‐growth relationship (Tables 4 and 5).

**TABLE 5:** Influence of behavior on life history (growth rate) across regions and developmental stages.

Growth rate including boldness (time to leave the shelter) as a predictor				
Predictors	SumSq (mean sq)	*F*‐value	*p*	df (Num_dem_)
Boldness	0.08 (0.08)	0.24	0.62	1_620.5_
Region	60.94 (15.23)***	46.71	**< 0.001**	1_41.8_
Stage	0.28 (0.28)	0.85	0.36	1_618.4_
Boldness × region	5.30 (1.32)**	4.06	**0.030**	1_620.4_
Boldness × stage	0.13 (0.13)	0.41	0.52	1_620.3_
Region × stage	2.90 (0.73)	2.21	0.065	1_618.4_
Boldness × region × stage	7.07 (1.77)***	5.42	**< 0.001**	1_620.3_

Random effects	Δ log‐lik	*χ* ^2^	*p*	df
Population	5.88***	11.8	**< 0.001**	1
*σ* ^2^	0.33			
ICC	0.07			
Observations	631			
Marginal *R* ^2^	0.651			

Growth rate including activity (mean speed) as predictor				
Predictors	SumSq (mean sq)	*F*‐value	*p*	df (Num_dem_)
Activity	0.13 (0.13)	0.38	0.54	1_463.0_
Region	25.33 (6.33)***	19.10	**< 0.001**	4_53.0_
Stage	0.64 (0.64)	1.93	0.16	1_463.1_
Activity × region	5.07 (1.29)**	3.83	**0.005**	4_462.2_
Activity × stage	0.13 (0.42)	0.39	0.53	1_462.9_
Region × stage	3.94 (0.99)*	2.97	**0.019**	4_462.9_
Activity × region × stage	5.04 (1.26)**	3.80	**0.005**	4_463.0_

Random effects	Δ log‐lik	*χ* ^2^	*p*	df
Population	3.6**	7.2	**0.071**	1
*σ* ^2^	0.33			
ICC	0.07			
Observations	489			
Marginal *R* ^2^	0.639			

Growth rate including exploration (mean distance to shelter) as predictor				
Predictors	SumSq (mean sq)	*F*‐value	*p*	df (Num_dem_)
Exploration	0.00 (0.0)	0.01	0.92	1_461.8_
Region	69.78 (17.45)***	51.71	**< 0.001**	4_11.5_
Stage	2.09 (2.09)*	6.21	**0.013**	1_463.6_
Exploration × region	0.68 (0.17)	0.50	0.74	4_462.0_
Exploration × stage	1.87 (1.87)*	5.54	**0.019**	1_463.5_
Region × stage	1.39 (0.35)	1.03	0.38	4_463.2_
Exploration × region × stage	1.50 (0.37)	1.11	0.35	4_463.3_

Random effects	Δ log‐lik	*χ* ^2^	*p*	df
Population	3.9***	7.7	**0.005**	1
Region	0.0	0	1	1
*σ* ^2^	0.34			
ICC	0.07			
Observations	489			
Marginal *R* ^2^	0.634			

*Note:* Results for statistical analyses evaluating growth rate in moor frog tadpoles and froglets originating from five different regions across a 1700 latitudinal gradient and raised in a common garden experiment (from South to North: Hanover (Germany), and Skåne, Uppsala, Umeå, Luleå (Sweden)). Growth rate models included the behavior of interest, region, developmental stage, and their interaction as predictors, and population of origin as a random intercept effect.

* *p* < 0.05.

** *p* < 0.01.

*** *p* < 0.001.

Growth/activity—In the genetic cluster model, no significant three‐way interaction or two‐way interactions were detected between activity, genetic cluster, and developmental stage (Figure 4B, Table 4). However, in the geographical region model, we found a significant three‐way interaction between activity, region, and developmental stage (Figure S2B; Table 5; Table S3).

This effect was primarily driven by a positive association between growth rate and activity in froglets sampled in Uppsala (estimate = 0.73 ± 0.25 SE, *p* = 0.032) and Luleå regions (estimate = 0.89 ± 0.35 SE, *p* = 0.059). Further post hoc analyses indicated that these associations in froglets differed significantly from other populations (e.g., Hanover vs. Uppsala contrast, and Luleå vs. Hanover, Skåne, and Umeå contrasts; see Figure S2A; Table S4). Additionally, in both Uppsala and Luleå, the positive association observed at the froglet stage contrasted with the absence of this relationship in tadpoles from these regions (Uppsala: ∆estimate_Tadpole vs. Froglet_ = −0.72 ± 0.26 SE: *t* = 2.82, *p* = 0.005; Luleå: ∆ estimate_Tadpole vs. Froglet_ = −2.46 ± 0.96 SE, *t* = 2.56, *p* = 0.011; Figure S2A; Tables S3 and S5). The random population effect in activity was significant in models incorporating a genetic cluster or region (Tables 4 and 5).

Growth/exploration—The relationship between growth rate and exploration was significantly influenced by genetic cluster and developmental stage. Specifically, we found a three‐way interaction between exploration, genetic cluster, and developmental stage, and a significant two‐way interaction between exploration and developmental stage (Table 4). In the North cluster, a negative relationship between exploration and growth rate was observed at the tadpole stage, but not at the froglet stage (Figure 4C; Table S2). In analyses conducted using geographical region, we also found an interaction between exploration and developmental stage (Figure S2B; Table 5; Table S3). This effect was largely driven by a reversal in the direction of this relationship in Luleå population samples, where exploration as tadpole was negatively associated with growth rate but positively associated with exploration as froglets (Tadpole: estimate = −0.15 ± 0.08 SE; Froglet: estimate = 0.21 ± 0.15 SE; ∆ estimate_Tadpole vs. Froglet_ = −0.36 ± 0.17 SE, *t* = −2.12, *p* = 0.034; Figure S2A; Table S5). As with boldness, in both models incorporating genetic cluster or geographic region, the random effect of population effect was significant, indicating additional population‐level variation in the exploration‐growth association (Tables 4 and 5).

## 4 Discussion

We found significant differences in proactivity levels between developmental stages and genetic clusters in *R. arvalis* , as well as along the geographical gradient. In particular, we found reversals in behavior across developmental stages in *R. arvalis* : boldness shifted from longest times to emerge in southern tadpoles to longest times to emerge in northern froglets; activity varied strongly among geographical regions in tadpoles, but was more similar in froglets; and exploration showed the opposite regional patterns—more similar across tadpole populations and more variation across froglet populations. We found no individual repeatability of boldness in repeated measurements taken within the tadpole stage, but boldness was significantly repeatable within the froglet stage. Assessment of cross‐stage repeatability using repeated measurements of the same individual at both the tadpole and froglet stages showed that activity was the only trait significantly consistent across stages, a pattern not observed for exploration and boldness in repeated measurements across stages. Finally, growth rate showed complex interactions with boldness, activity, and exploration, varying by genetic cluster, geographical region, and developmental stage.

### 4.1 Proactive Behaviors of Tadpoles Along the Latitudinal Gradient

Activity levels were higher in tadpoles from the North genetic cluster and in the three northernmost regions compared to the South genetic cluster and the two southernmost regions, respectively. Additionally, tadpoles from the two northernmost regions also showed higher activity levels than those from the latitudinally intermediate Uppsala region. Tadpoles from the northernmost region (Luleå) exhibited higher boldness than those from other regions, and tadpoles from the southernmost region (Hanover) showed lower boldness than those from other regions, including regions within the same genetic cluster. These results suggest that genetic clustering, arising from separate postglacial colonization events and subsequent demographic processes, together with contemporary regional selection, have shaped some proactive behaviors of this species at the tadpole stage. This is in line with previous studies showing a complex interplay between the effects of historical and contemporary processes on the genetic structure (Cortázar‐Chinarro et al. 2017; Cortazar‐Chinarro et al. 2018; Meyer‐Lucht et al. 2019; Rödin‐Mörch et al. 2019) and larval life history traits (Luquet et al. 2019) of *R. arvalis* populations along the latitudinal gradient. These findings align with the broader framework of life history theory, particularly the POLS hypothesis, which proposes that fast‐slow life history strategies coevolve with personality traits under environmental selection pressures (Réale et al. 2010). Our results in *R. arvalis* tadpoles provide partial support for this concept, particularly in the context of ectotherms facing strong seasonal constraints at early developmental stages. For instance, higher activity levels in northern tadpoles may reflect selection for faster growth and resource acquisition under shorter growing seasons. A similar pattern has been observed in antipredator responses across lizard species, where time‐limited environments select for risk‐prone behavior to maximize development under seasonal constraints (Samia et al. 2015). However, disentangling the contributions of historical genetic divergence and contemporary environmental selection remains challenging with our study design. This is so, as the observed differences in proactive behaviors between northern and southern populations could reflect either genetic clustering shaped by postglacial colonization events, environmental gradients associated with latitude, or an interaction between these two.

Our results further suggest that the expression of proactivity levels in *R. arvalis* tadpoles is trait‐specific and context‐dependent. Activity levels showed low variation among populations within genetic clusters, which may reflect genetic constraints on activity levels linked to colonization routes. On the contrary, boldness varied substantially among geographical regions, suggesting that boldness is more responsive to contemporary region‐specific environmental variation. This trait‐specific divergence in our latitudinal gradient underscores the need for caution when interpreting POLS predictions as uniform across behaviors (Dingemanse et al. 2010; Wolf and Weissing 2012), and contributes to the recent refinements of the POLS framework, emphasizing that personality traits may evolve independently and respond differently to historical events and ongoing environmental pressures (Royauté et al. 2018). Further experiments across generations, coupled with genomic analyses, should be essential to isolate the contribution of genetic and environmental plasticity to the expression of these behavioral traits.

### 4.2 Decoupling of Proactive Behaviors Across Ontogeny Along the Latitudinal Gradient

Interestingly, the proactive behavior of froglets along the gradient was almost the reverse of the tadpole behavior. The froglets in the North cluster exhibited reduced boldness and exploration compared to the South cluster, and the froglets in the two northern regions were shyer and explored less than the froglets in the other regions. There was no change in froglet activity along the gradient. This result aligns well with the extended POLS theory, which predicts that behavioral traits, such as boldness, may not remain consistently linked across life stages. In other words, behavioral tendencies along the proactive–reactive continuum can shift across ontogeny, leading to stage‐specific behavioral expression (Montiglio et al. 2018).

The observed decoupling is probably due to different selective pressures that amphibians face as they transition from aquatic to terrestrial environments after metamorphosis (Van Buskirk 2002; Relyea and Hoverman 2003; Orizaola and Brana 2005). Our results suggest that in high‐latitude populations, tadpoles may benefit from increased boldness and activity in aquatic environments, where rapid growth and resource acquisition are critical for survival, as the tadpoles have to metamorphose before the onset of winter (Laugen et al. 2003; Luquet et al. 2019; Orizaola et al. 2013). However, once living in terrestrial environments, the cost of being overly bold or explorative in harsher northern climates could outweigh the benefits. Specifically, in high‐latitude populations, time for foraging after metamorphosis is very short due to approaching winter, which may select for lower boldness and exploration activity. The highest level of exploration was found in the Hanover population, which experiences the longest season length along the gradient, while Luleå experiences the shortest, suggesting that this trait may be related to the time available for resource acquisition after metamorphosis. In juvenile *R. arvalis* , postmetamorphic terrestrial growth rates in the laboratory are lower in the northern compared to the southern populations (Chondrelli 2024), and studies on growth and survival patterns in Scandinavian *Rana* populations suggest that survival is higher, but maturation occurs later in the northernmost populations (Hjernquist et al. 2012; Räsänen et al. 2008; Söderman 2006). The results above suggest that time constraints for terrestrial growth and development may be relaxed along the latitudinal gradient as compared to the more stringent constraints faced by tadpoles. This shift may be a key contributor to the reversal of proactivity behaviors quantified in this study, reflecting changing ecological demands across ontogeny. Within the POLS framework, this pattern may indicate a reallocation of energy or risk‐taking strategies as individuals transition from a growth‐focused larval stage to a survival‐focused postmetamorphic stage, where behavioral traits like boldness or exploration may no longer align with fast‐growth strategies or may incur higher predation costs (Sih et al. 2004; Montiglio et al. 2018).

### 4.3 Relationship Between Proactive Behaviors and Larval Growth Along the Latitudinal Gradient

Developmental growth is an important life history trait in larval amphibians, and several studies have shown its divergence along climatic gradients (e.g., Berven and Gill 1983; Laurila et al. 2008; Orizaola et al. 2010). *R. arvalis* populations derived from the two post‐glaciation colonization routes differ significantly in larval growth rates, with populations from the northern cluster showing a higher growth rate (Luquet et al. 2019; Figure 4). In the present study, behavioral variation in tadpoles—mainly higher activity in the North cluster and greater boldness in the northernmost region—broadly aligns with expectations that proactive behaviors co‐evolve with faster larval growth. However, detailed relationships between proactive behaviors and larval growth showed a strong influence of both the genetic cluster and the developmental stage. Specifically, larval growth is positively associated with boldness in tadpoles from the South cluster but not in tadpoles from the North, whereas in froglets this association was reversed—positively associated with boldness in the North cluster but absent in the South.

At the regional scale, our findings further disentangle the role of historical processes and local adaptation in shaping growth‐behavior relationships. We observed positive associations between tadpole boldness and larval growth in Uppsala and Skåne, as well as between froglet activity and larval growth in Uppsala, but not in other regions. These results suggest that local environmental conditions have more strength in modulating such associations, as previously observed in insects (Golab et al. 2022).

As mentioned above, these different relationships across genetic clusters and among regions are likely driven by a combination of colonization history and climate‐induced time constraints, which have together shaped crucial shifts in metabolism and life‐history traits (Laurila et al. 2008; Orizaola et al. 2013; Caruso et al. 2020). In addition, ecological factors such as predation pressure and susceptibility to parasitic infections likely play crucial roles in shaping the contrasting relationships between life history and behavior in different geographical areas. For instance, as predation pressure and parasite diversity tend to decrease with increasing latitude (Roslin et al. 2017; Rohde 1999), the contrasting relationships between growth and proactivity across clusters may reflect the interplay of these ecological pressures. In the north cluster, where both predation and parasitism are expected to be lower, proactive behaviors may provide an advantage later in development, explaining the positive association between boldness and larval growth in alignment with previous findings that *R. arvalis* at higher latitudes are larger and older at reproduction (Söderman 2006). Conversely, in the south cluster, where predation and parasitism are greater, selection may favor proactivity earlier during the tadpole stage, enabling faster growth and providing an adaptive strategy that compensates for greater exposure to predators and infection. This divergence in selective pressures likely explains the inconsistencies observed between the genetic clusters, with local adaptation influencing how behavior and life‐history traits covariate across ontogeny and different regions along the gradient.

### 4.4 Consistency of Inter‐Individual Differences in Proactivity and Across Ontogeny

The repeatability of the behavior was assessed only at the time of emergence from the shelter. The froglets had a repeatable time to emerge from the shelter, but not the tadpoles. This suggests that the ontogenetic timing is important to consider in studies investigating the consistency of interindividual differences in behavior and assessing personalities. Indeed, studies across taxa support the idea that boldness often develops later in ontogeny, as observed in several vertebrate taxa where boldness may appear only after sexual maturation (Petelle et al. 2013; Polverino et al. 2016; Delval et al. 2020). Moreover, the environmental conditions experienced by previous generations and throughout the lifetime of an individual may also influence the emergence of personalities (e.g., Tariel et al. 2020; Płaskonka et al. 2024). In this study, the results suggest that the time to emerge from a shelter is a proxy of the bold‐shy axis of personality in *R. arvalis* and the late emergence of boldness during ontogeny. As it was not the main aim of this study, further assessment of within‐stage behavioral consistency is necessary before drawing firm conclusions about when personality emerges and how it is shaped by demographic, geographical, and local factors.

Repeatability estimates across life stages indicated no consistency in individuals' boldness and explorations over ontogeny, while activity was significantly repeatable across tadpole and froglet developmental stages. Previous studies across taxa have reported consistency in individual proactivity levels throughout life stages (reviewed by Koenig and Ousterhout 2018). In anurans, individual consistency in proactivity levels before and after metamorphosis has been observed in *Ambystoma maculatum* (Koenig and Ousterhout 2018), and in the activity and exploration levels of *Pelophylax ridibundus* from a single population from northern Germany (Wilson and Krause 2012). However, neither Płaskonka et al. (2024) in a single population of Polish *R. arvalis* nor Brodin et al. (2013) in several northern Swedish populations of *R. temporaria* found significant repeatability was found across life stages. Our results are in general agreement with previous studies, suggesting that repeatability across life stages can be weak and depend on environmental constraints. Indeed, our southernmost population exhibited the lowest changes in proactivity traits across life stages, while there was a tendency to gradually increase in change across life stages with increasing latitude. Together, these findings suggest that individual consistency in behavior across life stages may vary across taxa as a result of life history trade‐offs, where behavioral responses are shaped by ecological pressures at different developmental stages rather than by underlying genetic drivers of the fast‐slow continuum (Stott et al. 2024).

## 5 Conclusions

*R. arvalis* populations along a broad latitudinal gradient in northern Europe exhibit clear differences in proactivity levels, showing reversed behavioral patterns before and after metamorphosis. Our findings illustrate how demographic processes, climate‐induced developmental constraints, and ecological pressures interact to shape life‐history variation beyond the fast‐slow continuum. Moreover, they emphasize the importance of latitudinal gradient studies to elucidate evolutionary processes driving phenotypic variation in behavior among natural populations. Future research should investigate the role of temperature, predator diversity, and resource availability in shaping these behaviors and individual fitness. Furthermore, linking behavioral variation across developmental stages to specific genomic polymorphisms could provide valuable insights into evolutionary processes, historical selection, and the maintenance of behavioral diversity across regions.

## Author Contributions

**Maria Cortazar‐Chinarro:** conceptualization (supporting), writing – original draft (equal). **Alberto Corral‐Lopez:** conceptualization (supporting), data curation (lead), formal analysis (lead), investigation (supporting), visualization (lead), writing – original draft (equal). **Deike U. Lüdtke:** investigation (supporting), writing – review and editing (supporting). **Fredrik Tegnér:** investigation (supporting), writing – review and editing (supporting). **Emilien Luquet:** conceptualization (supporting), investigation (lead), methodology (lead), project administration (supporting), validation (supporting), writing – review and editing (equal). **Anssi Laurila:** conceptualization (lead), funding acquisition (lead), project administration (lead), resources (lead), supervision (lead), validation (lead), writing – review and editing (equal).

## Conflicts of Interest

The authors declare no conflicts of interest.

## Supporting information


**Data S1:** ece371945‐sup‐0001‐supinfo.pdf.

## Data Availability

The datasets and analysis code supporting this study are available via a Figshare repository (https://figshare.com/s/8a2a9b701f22265b496b) with DOI: https://doi.org/10.6084/m9.figshare.29278079.

## References

[ece371945-bib-0001] Alford, R. A. 1999. “Ecology: Resource Use, Competition, and Predation.” In Tadpoles: The Biology of Anuran Larvae, edited by R. W. McDiarmid and R. Altig , 240–278. University of Chicago Press.

[ece371945-bib-0002] Amat, I. , E. Desouhant , E. Gomes , J. Moreau , and K. Monceau . 2018. “Insect Personality: What Can We Learn From Metamorphosis?” Current Opinion in Insect Science 27: 46–51. 30025634 10.1016/j.cois.2018.02.014

[ece371945-bib-0003] Bakker, T. C. 1986. “Aggressiveness in Sticklebacks (*Gasterosteus aculeatus* L.): A Behaviour‐Genetic Study.” Behaviour 98: 1–144.

[ece371945-bib-0004] Bates, D. , M. Maechler , B. Bolker , et al. 2015. “Package ‘lme4’.” Convergence 12, no. 1: 2.

[ece371945-bib-0005] Bégué, L. , N. Tschirren , M. Peignier , B. Szabo , and E. Ringler . 2024. “Behavioural Consistency Across Metamorphosis in a Neotropical Poison Frog.” Evolutionary Ecology 38, no. 1: 157–174. 10.1007/s10682-023-10274-0 PMC7616151 38989472

[ece371945-bib-0085] Berven, K. A. , and D. E. Gill . 1983. “Interpreting Geographic Variation in Life‐History Traits.” American Zoologist 23, no. 1: 85–97.

[ece371945-bib-0006] Biro, P. A. , and J. A. Stamps . 2008. “Are Animal Personality Traits Linked to Life‐History Productivity?” Trends in Ecology & Evolution 23, no. 7: 361–368. 18501468 10.1016/j.tree.2008.04.003

[ece371945-bib-0007] Bouchard, T. J. , and J. C. Loehlin . 2001. “Genes, Evolution, and Personality.” Behavior Genetics 31, no. 3: 243–273. 11699599 10.1023/a:1012294324713

[ece371945-bib-0008] Branson, K. , A. A. Robie , J. Bender , P. Perona , and M. H. Dickinson . 2009. “High‐Throughput Ethomics in Large Groups of Drosophila.” Nature Methods 6, no. 6: 451–457. 19412169 10.1038/nmeth.1328 PMC2734963

[ece371945-bib-0009] Brodin, T. 2009. “Behavioral Syndrome Over the Boundaries of Life—Carryovers From Larvae to Adult Damselfly.” Behavioral Ecology 20, no. 1: 30–37.

[ece371945-bib-0010] Brodin, T. , M. I. Lind , M. K. Wiberg , and F. Johansson . 2013. “Personality Trait Differences Between Mainland and Island Populations in the Common Frog ( *Rana temporaria* ).” Behavioral Ecology and Sociobiology 67: 135–143.

[ece371945-bib-0011] Cabrera, D. , J. R. Nilsson , and B. D. Griffen . 2021. “The Development of Animal Personality Across Ontogeny: A Cross‐Species Review.” Animal Behaviour 173: 137–144.

[ece371945-bib-0012] Careau, V. , D. Thomas , M. M. Humphries , and D. Réale . 2008. “Energy Metabolism and Animal Personality.” Oikos 117, no. 5: 641–653.

[ece371945-bib-0082] Caruso, N. M. , C. L. Staudhammer , and L. J. Rissler . 2020. “A Demographic Approach to Understanding the Effects of Climate on Population Growth.” Oecologia 193, no. 4: 889–901. 32803340 10.1007/s00442-020-04731-8

[ece371945-bib-0013] Chang, C. C. , M. Moiron , A. Sánchez‐Tójar , P. T. Niemelä , and K. L. Laskowski . 2024. “What Is the Meta‐Analytic Evidence for Life‐History Trade‐Offs at the Genetic Level?” Ecology Letters 27, no. 1: e14354. 38115163 10.1111/ele.14354

[ece371945-bib-0014] Chondrelli, N. 2024. Thermal Mismatch in High Latitude Host‐Parasite Interactions (Doctoral Dissertation, Acta Universitatis Upsaliensis).

[ece371945-bib-0015] Cohen, J. 1985. Metamorphosis: Introduction, Usages, and Evolution, 1–19. Metamorphosis Oxford University Press.

[ece371945-bib-0017] Cortazar‐Chinarro, M. , Y. Meyer‐Lucht , A. Laurila , and J. Höglund . 2018. “Signatures of Historical Selection on MHC Reveal Different Selection Patterns in the Moor Frog ( *Rana arvalis* ).” Immunogenetics 70: 477–484. 29387920 10.1007/s00251-017-1051-1 PMC6006221

[ece371945-bib-0016] Cortázar‐Chinarro, M. , E. Z. Lattenkamp , Y. Meyer‐Lucht , E. Luquet , A. Laurila , and J. Höglund . 2017. “Drift, Selection, or Migration? Processes Affecting Genetic Differentiation and Variation Along a Latitudinal Gradient in an Amphibian.” BMC Evolutionary Biology 17, no. 1: 1–14. 28806900 10.1186/s12862-017-1022-z PMC5557520

[ece371945-bib-0018] Culumber, Z. W. 2022. “Variation in Behavioral Traits Across a Broad Latitudinal Gradient in a Livebearing Fish.” Evolutionary Ecology 36, no. 1: 75–91.

[ece371945-bib-0019] Delval, I. , M. Fernández‐Bolaños , and P. Izar . 2020. “A Longitudinal Assessment of Behavioral Development in Wild Capuchins: Personality Is Not Established in the First 3 Years.” American Journal of Primatology 82, no. 11: e23116. 32096276 10.1002/ajp.23116

[ece371945-bib-0020] Dingemanse, N. J. , and N. A. Dochtermann . 2013. “Quantifying Individual Variation in Behaviour: Mixed‐Effect Modelling Approaches.” Journal of Animal Ecology 82, no. 1: 39–54. 23171297 10.1111/1365-2656.12013

[ece371945-bib-0078] Dingemanse, N. J. , A. J. Kazem , D. Réale , and J. Wright . 2010. “Behavioural Reaction Norms: Animal Personality Meets Individual Plasticity.” Trends in Ecology & Evolution 25, no. 2: 81–89. 19748700 10.1016/j.tree.2009.07.013

[ece371945-bib-0021] Fischer, A. G. 1960. “Latitudinal Variations in Organic Diversity.” Evolution 14, no. 1: 64–81.

[ece371945-bib-0022] Foster, S. A. , M. A. Wund , and J. A. Baker . 2015. “Evolutionary Influences of Plastic Behavioral Responses Upon Environmental Challenges in an Adaptive Radiation.” Integrative and Comparative Biology 55, no. 3: 406–417. 26163679 10.1093/icb/icv083 PMC4642688

[ece371945-bib-0023] Gerlai, R. , and V. Csányi . 1990. “Genotype‐Environment Interaction and the Correlation Structure of Behavioral Elements in Paradise Fish ( *Macropodus opercularis* ).” Physiology & Behavior 47, no. 2: 343–356. 2333348 10.1016/0031-9384(90)90153-u

[ece371945-bib-0024] Golab, M. J. , S. Sniegula , and T. Brodin . 2022. “Cross‐Latitude Behavioural Axis in an Adult Damselfly Calopteryx Splendens (Harris, 1780).” Insects 13, no. 4: 342. 35447784 10.3390/insects13040342 PMC9027559

[ece371945-bib-0025] Gopal, A. C. , K. Alujević , and M. L. Logan . 2023. “Temperature and the Pace of Life.” Behavioral Ecology and Sociobiology 77, no. 5: 59.

[ece371945-bib-0026] Gosner, K. L. 1960. “A Simplified Table for Staging Anuran Embryos and Larvae With Notes on Identification.” Herpetologica 16, no. 3: 183–190.

[ece371945-bib-0027] Groothuis, T. G. , and C. Carere . 2005. “Avian Personalities: Characterization and Epigenesis.” Neuroscience and Biobehavioral Reviews 29, no. 1: 137–150. 15652261 10.1016/j.neubiorev.2004.06.010

[ece371945-bib-0028] Halekoh, U. , and S. Højsgaard . 2014. “A Kenward‐Roger Approximation and Parametric Bootstrap Methods for Tests in Linear Mixed Models – The R Package Pbkrtest.” Journal of Statistical Software 59, no. 9: 1–30.26917999

[ece371945-bib-0029] Healy, K. , T. H. Ezard , O. R. Jones , R. Salguero‐Gómez , and Y. M. Buckley . 2019. “Animal Life History Is Shaped by the Pace of Life and the Distribution of Age‐Specific Mortality and Reproduction.” Nature Ecology & Evolution 3, no. 8: 1217–1224. 31285573 10.1038/s41559-019-0938-7

[ece371945-bib-0030] Higgins, T. A. , R. C. Wilcox , R. R. Germain , and C. E. Tarwater . 2022. “Behavioral Traits Vary With Intrinsic Factors and Impact Local Survival in Song Sparrows (*Melospiza Melodia*).” Wilson Journal of Ornithology 134: 278–290.

[ece371945-bib-0081] Hjernquist, M. B. , F. Söderman , K. I. Jönsson , G. Herczeg , A. Laurila , and J. Merilä . 2012. “Seasonality Determines Patterns of Growth and Age Structure over a Geographic Gradient in an Ectothermic Vertebrate.” Oecologia 170, no. 3: 641–649. 22565493 10.1007/s00442-012-2338-4

[ece371945-bib-0031] Kelleher, S. R. , A. J. Silla , and P. G. Byrne . 2018. “Animal Personality and Behavioral Syndromes in Amphibians: A Review of the Evidence, Experimental Approaches, and Implications for Conservation.” Behavioral Ecology and Sociobiology 72, no. 5: 1–26.

[ece371945-bib-0032] Knopp, T. , and J. Merilä . 2009. “The Postglacial Recolonization of Northern Europe by *Rana arvalis* as Revealed by Microsatellite and Mitochondrial DNA Analyses.” Heredity 102, no. 2: 174–181. 18827835 10.1038/hdy.2008.91

[ece371945-bib-0033] Koenig, A. M. , and B. H. Ousterhout . 2018. “Behavioral Syndrome Persists Over Metamorphosis in a Pond‐Breeding Amphibian.” Behavioral Ecology and Sociobiology 72: 1–12.

[ece371945-bib-0034] Kuznetsova, A. , P. B. Brockhoff , and R. H. B. Christensen . 2017. “lmerTest Package: Tests in Linear Mixed Effects Models.” Journal of Statistical Software 82, no. 13: 1–26.

[ece371945-bib-0077] Laugen, A. T. , A. Laurila , K. Räsänen , and J. Merilä . 2003. “Latitudinal Countergradient Variation in the Common Frog (*Rana temporaria*) Development Rates–Evidence for Local Adaptation.” Journal of Evolutionary Biology 16, no. 5: 996–1005. 14635915 10.1046/j.1420-9101.2003.00560.x

[ece371945-bib-0035] Laurila, A. , B. Lindgren , and A. T. Laugen . 2008. “Antipredator Defenses Along a Latitudinal Gradient in *Rana temporaria* .” Ecology 89, no. 5: 1399–1413. 18543632 10.1890/07-1521.1

[ece371945-bib-0036] Laurila, A. , S. Pakkasmaa , and J. Merilä . 2006. “Population Divergence in Growth Rate and Antipredator Defenses in *Rana Arvalis* .” Oecologia 147: 585–595. 16323018 10.1007/s00442-005-0301-3

[ece371945-bib-0037] Lenth, R. 2016. “Least‐Squares Means: The R Package lsmeans.” Journal of Statistical Software 69, no. 1: 1–33. 10.18637/jss.v069.i01.

[ece371945-bib-0038] Luquet, E. , J. P. Léna , C. Miaud , and S. Plénet . 2015. “Phenotypic Divergence of the Common Toad ( *Bufo bufo* ) Along an Altitudinal Gradient: Evidence for Local Adaptation.” Heredity 114, no. 1: 69–79. 25074572 10.1038/hdy.2014.71 PMC4815602

[ece371945-bib-0039] Luquet, E. , P. Rödin Mörch , M. Cortázar‐Chinarro , Y. Meyer‐Lucht , J. Höglund , and A. Laurila . 2019. “Post‐Glacial Colonization Routes Coincide With a Life‐History Breakpoint Along a Latitudinal Gradient.” Journal of Evolutionary Biology 32, no. 4: 356–368. 30703260 10.1111/jeb.13419

[ece371945-bib-0040] Mazué, G. P. , F.‐X. Dechaume‐Moncharmont , and J.‐G. J. Godin . 2015. “Boldness–Exploration Behavioral Syndrome: Interfamily Variability and Repeatability of Personality Traits in the Young of the Convict Cichlid (Amatitlania Siquia).” Behavioral Ecology 26, no. 3: 900–908.

[ece371945-bib-0041] Meyer‐Lucht, Y. , E. Luquet , F. Jóhannesdóttir , et al. 2019. “Genetic Basis of Amphibian Larval Development Along a Latitudinal Gradient: Gene Diversity, Selection and Links With Phenotypic Variation in Transcription Factor C/EBP‐1.” Molecular Ecology 28, no. 11: 2786–2801. 31067349 10.1111/mec.15123

[ece371945-bib-0042] Mitchell, D. , E. T. Beatty , and P. K. Cox . 1977. “Behavioral Differences Between Two Populations of Wild Rats: Implications for Domestication Research.” Behavioral Biology 19, no. 2: 206–216. 557972 10.1016/s0091-6773(77)91494-8

[ece371945-bib-0044] Monceau, K. , J. Moreau , J. Richet , S. Motreuil , Y. Moret , and F. X. Dechaume‐Moncharmont . 2017. “Larval Personality Does Not Predict Adult Personality in a Holometabolous Insect.” Biological Journal of the Linnean Society 120, no. 4: 869–878.

[ece371945-bib-0045] Montiglio, P. O. , M. Dammhahn , G. D. Messier , and D. Réale . 2018. “The Pace‐Of‐Life Syndrome Revisited: The Role of Ecological Conditions and Natural History on the Slow‐Fast Continuum.” Behavioral Ecology and Sociobiology 72: 116.

[ece371945-bib-0046] Nakagawa, S. , and H. Schielzeth . 2010. “Repeatability for Gaussian and Non‐Gaussian Data: A Practical Guide for Biologists.” Biological Reviews 85, no. 4: 935–956. 20569253 10.1111/j.1469-185X.2010.00141.x

[ece371945-bib-0047] Orizaola, G. , and F. Brana . 2005. “Plasticity in Newt Metamorphosis: The Effect of Predation at Embryonic and Larval Stages.” Freshwater Biology 50, no. 3: 438–446.

[ece371945-bib-0048] Orizaola, G. , E. Dahl , A. G. Nicieza , and A. Laurila . 2013. “Larval Life History and Anti‐Predator Strategies Are Affected by Breeding Phenology in an Amphibian.” Oecologia 171: 873–881. 22976774 10.1007/s00442-012-2456-z

[ece371945-bib-0049] Orizaola, G. , M. Quintela , and A. Laurila . 2010. “Climatic Adaptation in an Isolated and Genetically Impoverished Amphibian Population.” Ecography 33, no. 4: 730–737.

[ece371945-bib-0050] Petelle, M. B. , D. E. McCoy , V. Alejandro , J. G. Martin , and D. T. Blumstein . 2013. “Development of Boldness and Docility in Yellow‐Bellied Marmots.” Animal Behaviour 86, no. 6: 1147–1154.

[ece371945-bib-0051] Płaskonka, B. , A. Zaborowska , A. Mikulski , and B. Pietrzak . 2024. “Predation Risk Experienced by Tadpoles Shapes Personalities Before but Not After Metamorphosis.” Ecology and Evolution 14, no. 11: e70532. 39539678 10.1002/ece3.70532 PMC11560291

[ece371945-bib-0052] Polverino, G. , C. Cigliano , S. Nakayama , and T. Mehner . 2016. “Emergence and Development of Personality Over the Ontogeny of Fish in Absence of Environmental Stress Factors.” Behavioral Ecology and Sociobiology 70: 2027–2037.

[ece371945-bib-0053] Poulin, R. , and T. L. F. Leung . 2011. “Latitudinal Gradient in the Taxonomic Composition of Parasite Communities.” Journal of Helminthology 85, no. 3: 228–233. 21070687 10.1017/S0022149X10000696

[ece371945-bib-0054] R Core Team . 2024. “R: A Language and Environment for Statistica Computing.” R Foundation for Statistical Computing, Vienna, Austria. https://www.R‐project.org/.

[ece371945-bib-0055] Räsänen, K. , F. Söderman , A. Laurila , and J. Merilä . 2008. “Geographic Variation in Maternal Investment: Acidity Affects Egg Size and Fecundity in *Rana arvalis* .” Ecology 89, no. 9: 2553–2562. 18831176 10.1890/07-0168.1

[ece371945-bib-0056] Réale, D. , D. Garant , M. M. Humphries , P. Bergeron , V. Careau , and P. O. Montiglio . 2010. “Personality and the Emergence of the Pace‐Of‐Life Syndrome Concept at the Population Level.” Philosophical Transactions of the Royal Society, B: Biological Sciences 365: 4051–4063. 10.1098/rstb.2010.0208 PMC2992747 21078657

[ece371945-bib-0057] Relyea, R. A. , and J. T. Hoverman . 2003. “The Impact of Larval Predators and Competitors on the Morphology and Fitness of Juvenile Treefrogs.” Oecologia 134: 596–604. 12647133 10.1007/s00442-002-1161-8

[ece371945-bib-0058] Rödin‐Mörch, P. , E. Luquet , Y. Meyer‐Lucht , A. Richter‐Boix , J. Höglund , and A. Laurila . 2019. “Latitudinal Divergence in a Widespread Amphibian: Contrasting Patterns of Neutral and Adaptive Genomic Variation.” Molecular Ecology 28, no. 12: 2996–3011. 31134695 10.1111/mec.15132

[ece371945-bib-0086] Rohde, K. 1999. “Latitudinal Gradients in Species Diversity and Rapoport's Rule Revisited: A Review of Recent Work and What Can Parasites Teach Us About the Causes of the Gradients?” Ecography 22, no. 6: 593–613.

[ece371945-bib-0059] Roslin, T. , B. Hardwick , V. Novotny , et al. 2017. “Higher Predation Risk for Insect Prey at Low Latitudes and Elevations.” Science 356, no. 6339: 742–744. 28522532 10.1126/science.aaj1631

[ece371945-bib-0080] Royauté, R. , M. A. Berdal , C. R. Garrison , and N. A. Dochtermann . 2018. “Paceless Life? A Meta‐Analysis of the Pace‐of‐Life Syndrome Hypothesis.” Behavioral Ecology and Sociobiology 72, no. 3: 64.

[ece371945-bib-0060] Samia, D. S. , A. P. Møller , D. T. Blumstein , T. Stankowich , and W. E. Cooper Jr. 2015. “Sex Differences in Lizard Escape Decisions Vary With Latitude, but Not Sexual Dimorphism.” Proceedings of the Royal Society B: Biological Sciences 282, no. 1805: 20150050. 10.1098/rspb.2015.0050 PMC4389619 25788595

[ece371945-bib-0061] Schemske, D. W. , G. G. Mittelbach , H. V. Cornell , J. M. Sobel , and K. Roy . 2009. “Is There a Latitudinal Gradient in the Importance of Biotic Interactions?” Annual Review of Ecology, Evolution, and Systematics 40: 245–269.

[ece371945-bib-0062] Sih, A. , A. M. Bell , J. C. Johnson , and R. E. Ziemba . 2004. “Behavioral Syndromes: An Integrative Overview.” Quarterly Review of Biology 79, no. 3: 241–277. 15529965 10.1086/422893

[ece371945-bib-0063] Sillero, N. , J. Campos , A. Bonardi , et al. 2014. “Updated Distribution and Biogeography of Amphibians and Reptiles of Europe.” Amphibia‐Reptilia 35, no. 1: 1–31.

[ece371945-bib-0064] Söderman, F. 2006. “Comparative Population Ecology in Moor Frogs With Particular Reference to Acidity (Doctoral Dissertation, Acta Universitatis Upsaliensis).”

[ece371945-bib-0065] Spiegel, O. , S. T. Leu , A. Sih , S. S. Godfrey , and C. M. Bull . 2015. “When the Going Gets Tough: Behavioural Type‐Dependent Space Use in the Sleepy Lizard Changes as the Season Dries.” Proceedings of the Royal Society B: Biological Sciences 282, no. 1819: 20151768. 10.1098/rspb.2015.1768 PMC4685807 26609082

[ece371945-bib-0066] Stamps, J. A. 2007. “Growth‐Mortality Tradeoffs and ‘Personality Traits’ in Animals.” Ecology Letters 10, no. 5: 355–363. 17498134 10.1111/j.1461-0248.2007.01034.x

[ece371945-bib-0067] Stearns, S. C. 1992. The Evolution of Life Histories. Oxford University Press.

[ece371945-bib-0068] Stoffel, M. A. , S. Nakagawa , and H. Schielzeth . 2017. “rptR: Repeatability Estimation and Variance Decomposition by Generalized Linear Mixed‐Effects Models.” Methods in Ecology and Evolution 8, no. 11: 1639–1644.

[ece371945-bib-0083] Stott, I. , R. Salguero‐Gómez , O. R. Jones , et al. 2024. “Life Histories Are Not Just Fast or Slow.” Trends in Ecology & Evolution 39, no. 9: 830–840. 39003192 10.1016/j.tree.2024.06.001

[ece371945-bib-0069] Tariel, J. , S. Plénet , and E. Luquet . 2020. “How Do Developmental and Parental Exposures to Predation Affect Personality and Immediate Behavioural Plasticity in the Snail *Physa acuta* ?” Proceedings of the Royal Society B 287, no. 1941: 20201761. 33352075 10.1098/rspb.2020.1761 PMC7779491

[ece371945-bib-0070] Therneau, T. M. , and T. Lumley . 2015. “Package ‘survival’.” R Top Doc 128, no. 10: 28–33.

[ece371945-bib-0071] Van Buskirk, J. 2002. “Phenotypic Lability and the Evolution of Predator‐Induced Plasticity in Tadpoles.” Evolution 56: 361–370. 11926504 10.1111/j.0014-3820.2002.tb01346.x

[ece371945-bib-0084] Van de Walle, J. , R. Fay , J. M. Gaillard , et al. 2023. “Individual Life Histories: Neither Slow nor Fast, Just Diverse.” Proceedings of the Royal Society B 290, no. 2002: 20230511. 37403509 10.1098/rspb.2023.0511 PMC10320331

[ece371945-bib-0072] Villegas‐Ríos, D. , D. Réale , C. Freitas , E. Moland , and E. M. Olsen . 2018. “Personalities Influence Spatial Responses to Environmental Fluctuations in Wild Fish.” Journal of Animal Ecology 87, no. 5: 1309–1319. 29888445 10.1111/1365-2656.12872 PMC6175438

[ece371945-bib-0073] Wilbur, H. M. 1980. “Complex Life Cycles.” Annual Review of Ecology and Systematics 11: 67–93.

[ece371945-bib-0074] Willig, M. R. , D. M. Kaufman , and R. D. Stevens . 2003. “Latitudinal Gradients of Biodiversity: Pattern, Process, Scale, and Synthesis.” Annual Review of Ecology, Evolution, and Systematics 34, no. 1: 273–309.

[ece371945-bib-0075] Wilson, A. D. , and J. Krause . 2012. “Personality and Metamorphosis: Is Behavioral Variation Consistent Across Ontogenetic Niche Shifts?” Behavioral Ecology 23, no. 6: 1316–1323.

[ece371945-bib-0076] Wilson, D. S. , A. B. Clark , K. Coleman , and T. Dearstyne . 1994. “Shyness and Boldness in Humans and Other Animals.” Trends in Ecology & Evolution 9, no. 11: 442–446. 10.1016/0169-5347(94)90134-1 21236920

[ece371945-bib-0079] Wolf, M. , and F. J. Weissing . 2012. “Animal Personalities: Consequences for Ecology and Evolution.” Trends in Ecology & Evolution 27, no. 8: 452–461. 22727728 10.1016/j.tree.2012.05.001

