# Supplementary material for: Metamorphosis Reverses the Behavioral Phenotype in Rana arvalis Along a Latitudinal Gradient
Source: Ecol Evol. 2025 Aug 13;15(8):e71945. doi: 10.1002/ece3.71945 (PMC12344277; doi:10.1002/ece3.71945)
Supplement: Supplementary file 1 — Data S1: ece371945‐sup‐0001‐supinfo.pdf. [file ECE3-15-e71945-s001.pdf]

Supplementary Information for:

Metamorphosis reverses the behavioral phenotype in *Rana arvalis* along a latitudinal gradient

List of contents:

<u>Figures</u>

- **Figure S1.** Consistency of individual behaviour across developmental stages.
- **Figure S2.** Association between behaviour and life history in moor frogs by region.

<u>Tables</u>

- **Table S1.** Independent contrasts of behavior models by developmental stage.
- **Table S2.** Independent contrasts of growth rate models by genetic cluster or by developmental stage.
- **Table S3.** Trends of growth rate and behavior association by region and developmental stage.
- **Table S4.** Independent contrasts of growth rate models by developmental stage.
- **Table S5.** Independent contrasts of growth rate models by region.

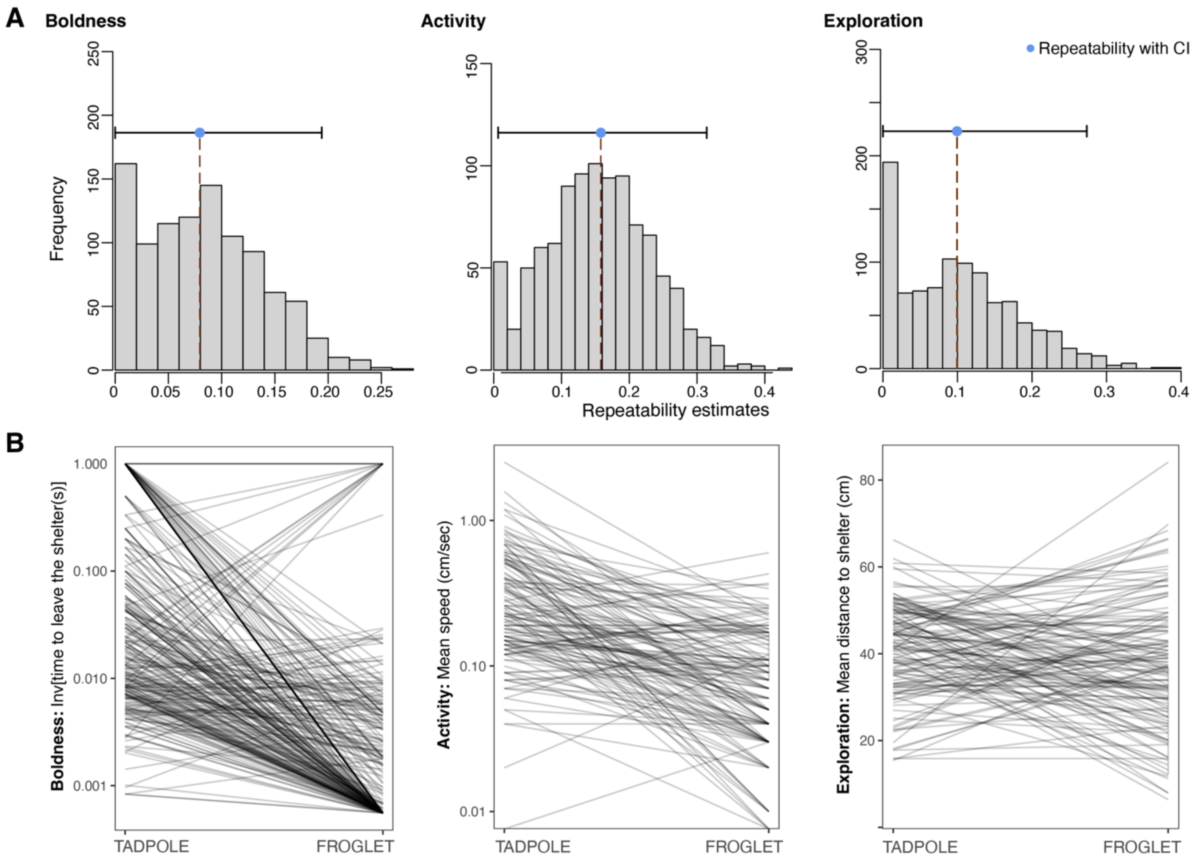

Figure S1. Consistency of individual behaviour across developmental stages. (A) Distribution of adjusted repeatability estimates of measurements in time to emerge from the shelter, activity and explorations taken as tadpole and froglet to each individual. Blue dot indicates mean adjusted repeatability with confidence intervals for each behavioural trait. (B) Individual differences in measurements of time to emerge from the shelter, activity and exploration taken to individuals as tadpole and froglet developmental stage. Lines represents measurements for each individual.

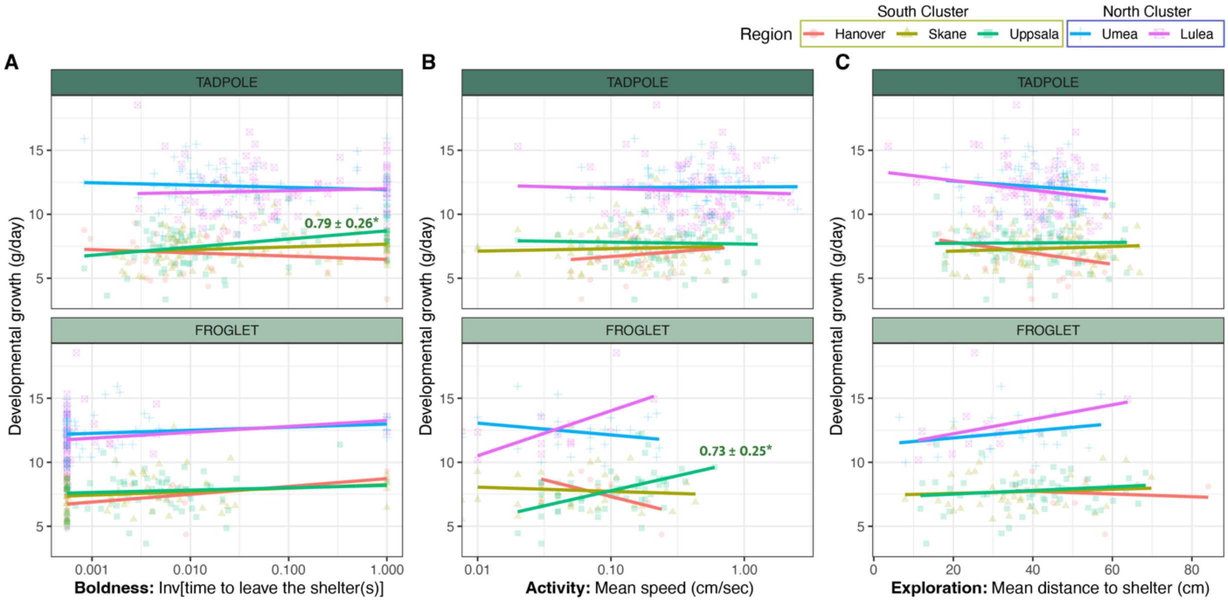

Figure S2. Association between behaviour and life history in moor frogs by region. Relationships between (A) boldness, (B) activity and (C) exploration and developmental growth (mass/time at Gosner stage 42) in moor frog tadpoles (top row) and froglets (bottom row) sampled in several regions across a 1700 latitudinal gradient and raised in a common garden experiment (South genetic cluster: Hanover (Germany), red dot; Skåne (Sweden), yellow triangle; Uppsala, green square); North genetic cluster: Umeå (Sweden), blue cross; Luleå (Sweden), pink crossed square). Trend lines are displayed for the relationship between each behaviour and developmental growth for values at the all regions. Text indicates estimated marginal means and standard error for significant trends in post-hoc contrasts of statistical models (see Table S5 for extended information on trends for each region).

Table S1. Independent contrasts of behavior models by developmental stage. Statistical contrasts between regions for significant interaction effects in models evaluating behavioral traits across tadpole and froglet stages in moor frogs originated from 5 different regions across a 1700 latitudinal gradient and raised in a common garden experiment. Contrasts were computed on estimated marginal means. Significant p-values adjusted for false discovery rate in bold.

Boldness: Time to leave the shelter

Dev.stage	*contrast*	*ratio*	*SE*	*df*	*z.ratio*	*p*
Tadpole	Hanover - Skåne	0.58	0.13	Inf	-2.40	**0.040**
	Hanover - Uppsala	0.69	0.15	Inf	-1.70	0.14
	Hanover - Umeå	0.74	0.18	Inf	-1.26	0.29
	Hanover - Luleå	0.38	0.09	Inf	-4.05	**<0.001**
	Skåne - Uppsala	1.17	0.19	Inf	0.98	0.36
	Skåne - Umeå	1.25	0.25	Inf	1.14	0.32
	Skåne - Luleå	0.65	0.12	Inf	-2.32	**0.040**
	Uppsala - Umeå	1.07	0.19	Inf	0.38	0.70
	Uppsala - Luleå	0.55	0.09	Inf	-3.45	**0.001**
	Umeå - Luleå	0.51	0.08	Inf	-4.15	**<0.001**
Froglet	Hanover - Skåne	1.27	0.34	Inf	0.88	0.42
	Hanover - Uppsala	0.98	0.26	Inf	-0.07	0.94
	Hanover - Umeå	2.01	0.54	Inf	2.58	**0.019**
	Hanover - Luleå	2.47	0.67	Inf	3.31	**0.002**
	Skåne - Uppsala	0.77	0.14	Inf	-1.36	0.25
	Skåne - Umeå	1.59	0.31	Inf	2.38	**0.028**
	Skåne - Luleå	1.95	0.38	Inf	3.40	**0.002**
	Uppsala - Umeå	2.05	0.37	Inf	3.94	**<0.001**
	Uppsala - Luleå	2.51	0.47	Inf	4.91	**<0.001**
	Umeå - Luleå	1.22	0.23	Inf	1.07	0.35

Activity: mean speed

Dev.stage	*contrast*	*estimate*	*SE*	*df*	*t-value*	*p*
Tadpole	Hanover - Skåne	0.03	0.07	9.33	0.50	0.69
	Hanover - Uppsala	-0.11	0.07	9.47	-1.71	0.15

	Hanover - Umeå	-0.21	0.07	11.01	-3.09	**0.022**
	Hanover - Luleå	-0.22	0.07	10.35	-3.24	**0.022**
	Skåne - Uppsala	-0.15	0.05	9.54	-3.13	**0.022**
	Skåne - Umeå	-0.25	0.05	12.72	-4.85	**0.002**
	Skåne - Luleå	-0.26	0.05	11.33	-5.16	**0.002**
	Uppsala - Umeå	-0.10	0.05	11.01	-2.03	0.095
	Uppsala - Luleå	-0.11	0.05	10.03	-2.23	0.081
	Umeå - Luleå	-0.00	0.05	9.50	-0.15	0.87
Froglet	Hanover - Skåne	0.01	0.09	29.03	0.13	0.90
	Hanover - Uppsala	-0.05	0.09	26.55	-0.53	0.78
	Hanover - Umeå	0.04	0.09	32.00	0.40	0.78
	Hanover - Luleå	0.09	0.10	44.57	0.92	0.73
	Skåne - Uppsala	-0.06	0.06	19.37	-1.02	0.73
	Skåne - Umeå	0.03	0.06	29.64	0.40	0.78
	Skåne - Luleå	0.08	0.07	55.29	1.06	0.73
	Uppsala - Umeå	0.08	0.06	22.89	1.40	0.73
	Uppsala - Luleå	0.14	0.07	47.43	1.92	0.61
	Umeå - Luleå	0.06	0.08	60.42	0.71	0.77

Exploration: mean distance to shelter

Dev.stage	*contrast*	*estimate*	*SE*	*df*	*t-value*	*p*
Tadpole	Hanover - Skåne	-2.40	2.49	12.88	-0.96	0.507
	Hanover - Uppsala	2.60	2.50	12.95	1.03	0.507
	Hanover - Umeå	-0.62	2.52	13.33	-0.24	0.836
	Hanover - Luleå	0.52	2.49	12.81	0.21	0.836
	Skåne - Uppsala	5.00	1.78	13.23	2.80	0.146
	Skåne - Umeå	1.77	1.80	14.01	0.98	0.507
	Skåne - Luleå	2.92	1.77	12.94	1.65	0.407
	Uppsala - Umeå	-3.22	1.81	14.16	-1.78	0.407
	Uppsala - Luleå	-2.07	1.77	13.09	-1.16	0.507
	Umeå - Luleå	1.15	1.80	13.86	0.64	0.664

Froglet	Hanover - Skåne	15.86	4.40	104.87	3.60	**0.001**
	Hanover - Uppsala	12.06	4.25	94.08	2.83	**0.011**
	Hanover - Umeå	18.33	4.58	118.61	4.00	**0.001**
	Hanover - Luleå	24.97	5.06	153.78	4.92	**<0.001**
	Skåne - Uppsala	-3.79	2.51	48.77	-1.51	0.153
	Skåne - Umeå	2.47	3.03	93.08	0.81	0.417
	Skåne - Luleå	9.11	3.78	161.41	2.44	**0.026**
	Uppsala - Umeå	6.27	2.81	72.59	2.22	**0.041**
	Uppsala - Luleå	12.91	3.55	141.14	3.63	**0.001**
	Umeå - Luleå	6.638	3.938	186.173	1.686	0.117

Table S2. Independent contrasts of growth rate models by genetic cluster or by developmental stage. Statistical contrasts of significant interaction effects between clusters or developmental stages in models evaluating growth rate in moor frog tadpoles and froglets divided in two genetic clusters based on their known colonization routes (South genetic cluster: Hanover (Germany), Skåne (Sweden), Uppsala), North genetic cluster: Umeå (Sweden), Luleå (Sweden)). Contrasts were computed on estimated marginal means. Significant p-values adjusted for false discovery rate in bold.

Growth rate including boldness (time to leave shelter shelter) as predictor

contrast	*Variable*	*estimate*	*SE*	*df*	*t.ratio*	*p.value*
North - South	Tadpole	0.745*	0.280	620	2.658	**0.008**
	Froglet	-0.070	0.073	618	-0.964	0.336
Tadpole - Froglet	North	0.512*	0.235	621	2.180	**0.030**
	South	-0.304	0.170	617	-1.792	0.074

Growth rate including exploration (mean distance to shelter) as predictor

contrast	*Variable*	*estimate*	*SE*	*df*	*t.ratio*	*p.value*
North - South	Tadpole	-0.117	0.075	476	-1.565	**0**.12
	Froglet	0.094	0.100	474	0.931	0.35
Tadpole - Froglet	North	-0.263*	0.108	475	-2.438	**0.015**
	South	-0.052	0.064	474	-0.811	0.42

Table S3. Trends of growth rate and behavior association by region and developmental stage. Pairwise contrasts of slopes for the relationship between behaviors and developmental growth across regions and develomental stages in moor frog tadpoles and froglets originating from five different regions across a 1700 latitudinal gradient and raised in a common garden experiment (Hanover (Germany), Skåne (Sweden), Uppsala), Umeå (Sweden), Luleå (Sweden)). Contrasts were computed on estimated marginal means. Significant p-values adjusted for false discovery rate in bold.

Boldness (time to leave shelter shelter)

Dev. stage	*region*	*Trend*	*SE*	*df*	*t.ratio*	*p.value*
Tadpole	Hanover	-0.376	0.268	603.004	-1.406	0.229
	Skåne	0.886	0.351	605.509	2.526	0.059
	Uppsala	0.790*	0.263	603.603	3.009	**0.027**
	Umeå	-0.282	0.240	607.843	-1.176	0.267
	Luleå	-1.089	0.743	607.078	-1.466	0.229
Froglet	Germany	0.142	0.111	603.004	1.275	0.254
	Skåne	0.115	0.066	606.007	1.755	0.199
	Uppsala	-0.001	0.068	603.582	-0.008	0.994
	Umeå	0.122	0.078	607.639	1.562	0.229
	Luleå	0.163	0.092	604.468	1.772	0.199

Activity (mean speed)

Dev. Stage	*region*	*Trend*	*SE*	*df*	*t.ratio*	*p.value*
Tadpole	Hanover	-0.031	0.257	461.015	-0.121	0.944
	Skåne	0.037	0.141	465.095	0.260	0.944
	Uppsala	0.005	0.071	468.702	0.070	0.944
	Umeå	-0.023	0.054	468.252	-0.433	0.944
	Luleå	0.007	0.052	461.557	0.140	0.944
Froglet	Germany	-1.793	0.931	461.015	-1.927	0.182
	Skåne	-0.126	0.317	462.168	-0.397	0.944
	Uppsala	0.727*	0.246	463.509	2.959	**0.032**
	Umeå	-0.320	0.567	461.158	-0.565	0.944

	Luleå	2.468	0.959	464.822	2.572	0.052

Exploration (mean distance to shelter)

Dev. Stage	*region*	*Trend*	*SE*	*df*	*t.ratio*	*p.value*
Tadpole	Hanover	-0.191	0.124	461.016	-1.541	0.493
	Skåne	0.035	0.064	463.134	0.549	0.834
	Uppsala	0.003	0.066	461.641	0.038	0.970
	Umeå	-0.112	0.096	466.914	-1.168	0.585
	Luleå	-0.147	0.081	464.300	-1.808	0.493
Froglet	Germany	-0.047	0.163	461.016	-0.286	0.861
	Skåne	0.031	0.083	461.396	0.375	0.861
	Uppsala	0.058	0.066	461.168	0.872	0.639
	Umeå	0.122	0.116	461.836	1.054	0.585
	Luleå	0.215	0.149	463.506	1.449	0.493

Table S4. Independent contrasts of growth rate models by developmental stage. Statistical contrasts of significant interaction effects between regions in models evaluating growth rate in moor frog tadpoles and froglets originating from five different regions across a 1700 latitudinal gradient and raised in a common garden experiment (Hanover (Germany), Skåne (Sweden), Uppsala), Umeå (Sweden), Luleå (Sweden)). Contrasts were computed on estimated marginal means. Significant p-values adjusted for false discovery rate in bold.

Growth rate including boldness (time to leave shelter shelter) as predictor

Dev.stage	*contrast*	*estimate*	*SE*	*df*	*t.ratio*	*p.value*
Tadpole	Hanover - Skåne	1.262	0.441	604.639	2.861	**0.015**
	Hanover - Uppsala	1.167	0.375	603.301	3.111	**0.013**
	Hanover - Umeå	0.094	0.359	605.423	0.262	0.828
	Hanover - Luleå	-0.713	0.790	606.681	-0.903	0.459
	Skåne - Uppsala	-0.095	0.438	604.855	-0.217	0.828
	Skåne - Umeå	-1.168	0.425	606.321	-2.749	**0.015**
	Skåne - Luleå	-1.975	0.821	606.811	-2.404	**0.029**
	Uppsala - Umeå	-1.072	0.356	605.743	-3.015	**0.013**
	Uppsala - Luleå	-1.879	0.788	606.744	-2.385	**0.029**
	Umeå - Luleå	-0.807	0.781	607.154	-1.034	0.431
Froglet	Hanover - Skåne	-0.027	0.129	603.843	-0.206	0.950
	Hanover - Uppsala	-0.142	0.131	603.164	-1.090	0.690
	Hanover - Umeå	-0.020	0.136	604.736	-0.149	0.950
	Hanover - Luleå	0.021	0.144	603.614	0.145	0.950
	Skåne - Uppsala	-0.116	0.095	604.802	-1.221	0.690
	Skåne - Umeå	0.006	0.102	606.998	0.062	0.950
	Skåne - Luleå	0.048	0.113	605.010	0.421	0.950
	Uppsala - Umeå	0.122	0.104	606.059	1.178	0.690
	Uppsala - Luleå	0.163	0.114	604.158	1.426	0.690
	Umeå - Luleå	0.041	0.120	605.916	0.342	0.950

Growth rate including activity (mean speed) as predictor

Dev. stage	*contrast*	*estimate*	*SE*	*df*	*t.ratio*	*p.value*
Tadpole	Hanover - Skåne	-0.068	0.293	462.077	-0.231	0.979
	Hanover - Uppsala	-0.036	0.267	461.892	-0.136	0.979

	Hanover - Umeå	-0.008	0.263	461.840	-0.030	0.979
	Hanover - Luleå	-0.038	0.262	461.037	-0.147	0.979
	Skåne - Uppsala	0.032	0.158	466.156	0.200	0.979
	Skåne - Umeå	0.060	0.151	466.373	0.398	0.979
	Skåne - Luleå	0.029	0.150	464.726	0.195	0.979
	Uppsala - Umeå	0.028	0.089	468.999	0.317	0.979
	Uppsala - Luleå	-0.002	0.088	467.273	-0.026	0.979
	Umeå - Luleå	-0.031	0.075	468.194	-0.409	0.979
Froglet	Hanover - Skåne	-1.667	0.983	461.139	-1.696	0.113
	Hanover - Uppsala	-2.520	0.963	461.191	-2.618	**0.032**
	Hanover - Umeå	-1.473	1.090	461.054	-1.351	0.197
	Hanover - Luleå	-4.261	1.337	463.121	-3.188	**0.015**
	Skåne - Uppsala	-0.853	0.401	462.688	-2.127	0.068
	Skåne - Umeå	0.194	0.649	461.404	0.300	0.765
	Skåne - Luleå	-2.594	1.010	464.588	-2.567	**0.032**
	Uppsala - Umeå	1.047	0.618	461.556	1.695	0.113
	Uppsala - Luleå	-1.741	0.990	464.746	-1.758	0.113
	Umeå - Luleå	-2.788	1.114	463.978	-2.502	**0.032**

Table S5. Independent contrasts of growth rate models by region. Statistical contrasts of significant interaction effects between developmental stages in models evaluating growth rate in moor frog tadpoles and froglets originating from five different regions across a 1700 latitudinal gradient and raised in a common garden experiment (Hanover (Germany), Skåne (Sweden), Uppsala), Umeå (Sweden), Luleå (Sweden)). Contrasts were computed on estimated marginal means. Significant p-values adjusted for false discovery rate in bold.

Growth rate including boldness (time to leave shelter shelter) as predictor

contrast	*region*	*estimate*	*SE*	*df*	*t.ratio*	*p.value*
Tadpole - Froglet	Hanover	-0.518	0.290	603.004	-1.788	0.074
	Skåne	0.770	0.356	604.822	2.163	**0.031**
	Uppsala	0.791	0.272	603.876	2.912	**0.004**
	Umeå	-0.404	0.251	605.675	-1.609	0.108
	Luleå	-1.252	0.748	606.909	-1.673	0.095

Growth rate including activity (mean speed) as predictor

contrast	*region*	*estimate*	*SE*	*df*	*t.ratio*	*p.value*
Tadpole - Froglet	Hanover	1.762	0.966	461.015	1.825	0.069
	Skåne	0.163	0.348	464.109	0.467	0.641
	Uppsala	-0.722	0.256	464.031	-2.822	**0.005**
	Umeå	0.297	0.569	461.078	0.522	0.602
	Luleå	-2.460	0.961	464.766	-2.561	**0.011**

Growth rate including exploration (mean distance to shelter) as predictor

contrast	*region*	*estimate*	*SE*	*df*	*t.ratio*	*p.value*
Tadpole - Froglet	Hanover	-0.144	0.205	461.016	-0.704	0.482
	Skåne	0.004	0.104	461.621	0.038	0.970
	Uppsala	-0.055	0.094	461.393	-0.590	0.555
	Umeå	-0.234	0.151	465.553	-1.552	0.121
	Luleå	-0.362	0.170	465.554	-2.129	**0.034**
